# Viral uptake and pathophysiology of the lung endothelial cells in age‐associated severe SARS‐CoV‐2 infection models

**DOI:** 10.1111/acel.14050

**Published:** 2023-12-14

**Authors:** Takuya Tsumita, Ryo Takeda, Nako Maishi, Yasuhiro Hida, Michihito Sasaki, Yasuko Orba, Akihiko Sato, Shinsuke Toba, Wataru Ito, Takahito Teshirogi, Yuya Sakurai, Tomohiro Iba, Hisamichi Naito, Hitoshi Ando, Haruhisa Watanabe, Amane Mizuno, Toshiki Nakanishi, Aya Matsuda, Ren Zixiao, Ji‐Won Lee, Tadahiro Iimura, Hirofumi Sawa, Kyoko Hida

**Affiliations:** ^1^ Department of Vascular Biology and Molecular Pathology, Faculty and Graduate School of Dental Medicine Hokkaido University Sapporo Japan; ^2^ Department of Oral Diagnosis and Medicine, Faculty and Graduate School of Dental Medicine Hokkaido University Sapporo Japan; ^3^ Department of Advanced Robotic and Endoscopic Surgery Fujita Health University Toyoake Japan; ^4^ Division of Molecular Pathobiology, International Institute for Zoonosis Control Hokkaido University Sapporo Japan; ^5^ International Collaboration Unit, International Institute for Zoonosis Control Hokkaido University Sapporo Japan; ^6^ Drug Discovery and Disease Research Laboratory Shionogi and Co., Ltd. Osaka Japan; ^7^ Department of Oral and Maxillofacial Surgery, Faculty and Graduate School of Dental Medicine Hokkaido University Sapporo Japan; ^8^ Department of Dental Anesthesiology, Faculty and Graduate School of Dental Medicine Hokkaido University Sapporo Japan; ^9^ Department of Vascular Physiology, Graduate School of Medical Sciences Kanazawa University Kanazawa Japan; ^10^ Department of Cellular and Molecular Function Analysis, Graduate School of Medical Sciences Kanazawa University Kanazawa Japan; ^11^ Department of Pharmacology, Faculty and Graduate School of Dental Medicine Hokkaido University Sapporo Japan; ^12^ One Health Research Center Hokkaido University Sapporo Japan; ^13^ Institute for Vaccine Research and Development Hokkaido University Sapporo Japan

**Keywords:** endothelial cells, mouse model, pathology, SARS‐CoV‐2, thrombosis

## Abstract

Thrombosis is the major cause of death in severe acute respiratory syndrome coronavirus 2 (SARS‐CoV‐2) infection, and the pathology of vascular endothelial cells (ECs) has received much attention. Although there is evidence of the infection of ECs in human autopsy tissues, their detailed pathophysiology remains unclear due to the lack of animal model to study it. We used a mouse‐adapted SARS‐CoV‐2 virus strain in young and mid‐aged mice. Only mid‐aged mice developed fatal pneumonia with thrombosis. Pulmonary ECs were isolated from these infected mice and RNA‐Seq was performed. The pulmonary EC transcriptome revealed that significantly higher levels of viral genes were detected in ECs from mid‐aged mice with upregulation of viral response genes such as DDX58 and IRF7. In addition, the thrombogenesis‐related genes encoding PLAT, PF4, F3 PAI‐1, and P‐selectin were upregulated. In addition, the inflammation‐related molecules such as CXCL2 and CXCL10 were upregulated in the mid‐aged ECs upon viral infection. Our mouse model demonstrated that SARS‐CoV‐2 virus entry into aged vascular ECs upregulated thrombogenesis and inflammation‐related genes and led to fatal pneumonia with thrombosis. Current results of EC transcriptome showed that EC uptake virus and become thrombogenic by activating neutrophils and platelets in the aged mice, suggesting age‐associated EC response as a novel finding in human severe COVID‐19.

## Graphical abstract

Although viral infection of ECs has been suggested as a mechanism for thrombosis in COVID‐19, their detailed pathophysiology remains unclear. We used a mouse‐adapted SARS‐CoV‐2 virus strain in young and mid‐aged mice. Only mid‐aged mice developed fatal pneumonia with thrombosis. Pulmonary ECs were isolated from these infected mice and RNA‐Seq was performed. The EC transcriptome data demonstrated SARS‐CoV‐2 uptake into ECs and a distinct thrombopathy phenotype in the mid‐aged mice, but not in the young mice.

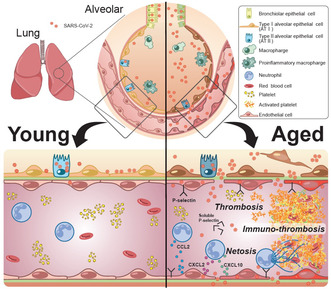

**Abbreviations**
ACE2: angiotensin converting enzyme II
BSL‐3: biosafety level 3
CCL2: C‐C motif chemokine ligand 2
CD: cluster of differentiation
CXCL10: C‐X‐C motif chemokine ligand 10
CXCL2: C‐X‐C motif chemokine ligand 2
DDX58: DEAD box protein 58
dpi: days post infection
ECs: endothelial cells
EIF2AK2: eukaryotic translation initiation factor 2 alpha kinase 2
ELISA: enzyme‐linked immunosorbent assay
F3: coagulation factor III
FACS: fluorescence‐activated cell sorting
Fbn1: fibrillin 1
FPKM: fragments per kilobase of transcript per million mapped reads
GSEA: gene set enrichment analysis
H&E: hematoxylin and eosin
HIF‐1a: hypoxia‐inducible factor 1a
HUVECs: human umbilical vein endothelial cells
ICAM‐1: intercellular adhesion molecule 1
IFN: interferon
IL‐1β: interleukin‐1β
IL‐6: interleukin‐6
IPA: ingenuity pathway analysis
IRF: interferon regulated factor
JAK: Janus kinases
MACS: magnetic‐activated cell sorting
MA‐P10: mouse‐adapted SARS‐CoV‐2 strain
MOI: multiplicity of infection
MPO: myeloperoxidase
N protein: nucleocapsid protein
NETs: neutrophil extracellular traps
NRP1: neuropilin 1
PAI‐1: plasminogen activator inhibitor 1
Pf4: platelet factor 4
PI: propidium iodide
PLAT: plasminogen activator, tissue type
RIG‐I: retinoic acid‐inducible gene‐I
SARS‐CoV‐2: severe acute respiratory syndrome coronavirus 2
STAT: signal transducer and activator of transcription
TF: tissue factor
Tlr4: toll like receptor 4
TMEM106B: transmembrane protein 106B
TMPRSS2: transmembrane serine protease 2
VCAM‐1: vascular cell adhesion molecule 1
VEGF: vascular endothelial growth factor
vWF: von Willebrand factor

## 1 INTRODUCTION

More than 3 years have passed since the onset of the new coronavirus pandemic, and the mortality rate of infected patients worldwide has declined from more than 7% at the early stages to about 1% in 2022 (https://coronavirus.jhu.edu/map.html). Nevertheless, the emergence of variants of interest and variants of concern (https://www.who.int/activities/tracking‐SARS‐CoV‐2‐variants) has increased the total number of infected people. Despite the development of vaccines, antiviral drugs, neutralizing antibodies, and anti‐inflammatory drugs, the burden on medical care has not decreased yet (Mogharab et al., 2022). People with COVID‐19 reportedly have had a wide range of symptoms, which range from mild symptoms (e.g., fever, cough, and sore throat) to severe illness (e.g., trouble breathing and persistent pain or pressure in the chest; https://www.cdc.gov/coronavirus/2019‐ncov/symptoms‐testing/symptoms.html).

Thromboembolism has received more attention as a causative mechanism of sudden death during the convalescence period, prolonged critical care, and multiple‐organ failure. Thromboembolism has been observed in as much as 40% of all COVID‐19 cases (Helms et al., 2020).

The most likely scenario of thromboembolism associated with the COVID‐19 infection may be related to the activation of the extrinsic coagulation pathway, through the cytokine storms associated with pneumonia, thus causing severe vascular endothelial cell (EC) damages. As COVID‐19 vasculitis is uniquely more extensive and more severe than that in the other pneumonia (McGonagle et al., 2021), other critical mechanisms besides the cytokine storm may be postulated.

Anatomically, the pulmonary vessels that underlay the alveolar epithelium through the basement membrane, and the possible viral infection reaching the alveolar ECs associated with COVID‐19 severity have been extensively discussed (Ackermann et al., 2020; Milross et al., 2022; Varga et al., 2020). In fact, the electron microscopic images for SARS‐CoV‐2 in the autopsy specimens revealed the existence of viral particles in ECs (Varga et al., 2020). Several previous review articles have discussed SARS‐CoV‐2 infection in ECs as a possible mechanism of severe COVID‐19 (Gu et al., 2021; Smadja et al., 2021). Moreover, it has been shown that the abnormal morphology of the capillary ECs, the accumulation of numerous immune cells around the blood vessels (Varga et al., 2020), and the gene signature of the enhanced angiogenic signals in COVID‐19 vary significantly from those in other viral infections such as influenza (Ackermann et al., 2020). Therefore, the specificity of the vascular endothelial pathology in COVID‐19 (endotheliopathy) has attracted much attention. By contrast, several previous reports proposed the nonexistence of in vivo SARS‐CoV‐2 infection of ECs (Schimmel et al., 2021), which has led to controversy and even to more skeptical views regarding viral infection of the pulmonary ECs.

One of the reasons for the lack of progress in elucidating the endothelial pathology in COVID‐19 is that the mouse models of severe COVID‐19, which are indispensable research tools, have not been commonly used until recently, and the common cultured ECs used for the research have low expression of the SARS‐CoV‐2 receptor, angiotensin‐converting enzyme II (ACE2). Additionally, it has been reported that the SARS‐CoV‐2 does not infect ECs under in vitro culture conditions and that the virus infects ECs in 3D co‐cultured with the epithelial cells only, which have hindered the analysis using the common ECs (McCracken et al., 2021; Schimmel et al., 2021). Recently, transcriptome analysis has been reported to be used at a single cell level of the lung tissue, which is dissected in an autopsy of human critically ill COVID‐19 patients (Delorey et al., 2021). SARS‐CoV‐2‐infected population has been observed previously in ECs; however, the number of infected ECs was small, and the sample was a mixture of multiple patient tissues. Therefore, the involvement of genes in the endothelial pathology of patients with severe COVID‐19 remains unclear. An animal model should be used when investigating the molecular details of pathogenesis involved in the thrombosis that is associated with SARS‐CoV‐2 infection. Hamsters are often utilized as animal models for COVID‐19; however, thrombosis and lung alveolar damage are not observed in this animal model. Initially, the hACE2–transgenic mice were applied for SARS‐CoV‐2 research, but these mice mainly suffer from fatal neurological symptoms (Huang et al., 2021). Recently, the mouse‐adapted SARS‐CoV‐2 strains has been established for investigating the host interaction that is caused by SARS‐CoV‐2 infection (Iwata‐Yoshikawa et al., 2022; Leist et al., 2020; Sun et al., 2021). In this study, a mouse‐adapted SARS‐CoV‐2 strain (MA‐P10) was established. SPF 30‐ to 50‐week‐old female BALB/c mice (BALB/cAJcl, CLEA Japan) were inoculated intranasally with the SARS‐CoV‐2 WK‐521 ancestral strain. Three days after infection, the virus in the supernatant of lung homogenate was intranasally injected into another BALB/c mouse. This adaptation process was performed 10 times. MA‐P10 harbors a G498H substitution in the spike protein and can efficiently replicate in normal mice and cause fatal pneumonia in mid‐aged mice (30–50 weeks old) only (Uemura et al., 2023). Therefore, MA‐P10‐infected mid‐aged mice are expected to serve as models for researching severe COVID‐19 in humans. ECs have been recognized to undergo various in vivo phenotypic changes, depending on the surrounding microenvironment. We have isolated ECs from the various malignant tumors (Hida et al., 2004) and have recorded their heterogeneity that is caused by the microenvironment (Ohga et al., 2012). Moreover, ECs have been observed to affect the surrounding cells through the secretion of a cytokine or damage‐associated molecular patterns (Cong et al., 2021; Maishi et al., 2016).

In this study, the transcriptomes of the pulmonary ECs isolated from the lung tissues of the MA‐P10‐infected mice have been analyzed using RNA sequencing (RNA‐Seq), to demonstrate the involvement of ECs in the pathogenesis of severe COVID‐19.

## 2 RESULTS

### 2.1 Mouse‐adapted SARS‐CoV‐2 infection causes fatal pneumonia in the mid‐aged mice

**FIGURE 1:**
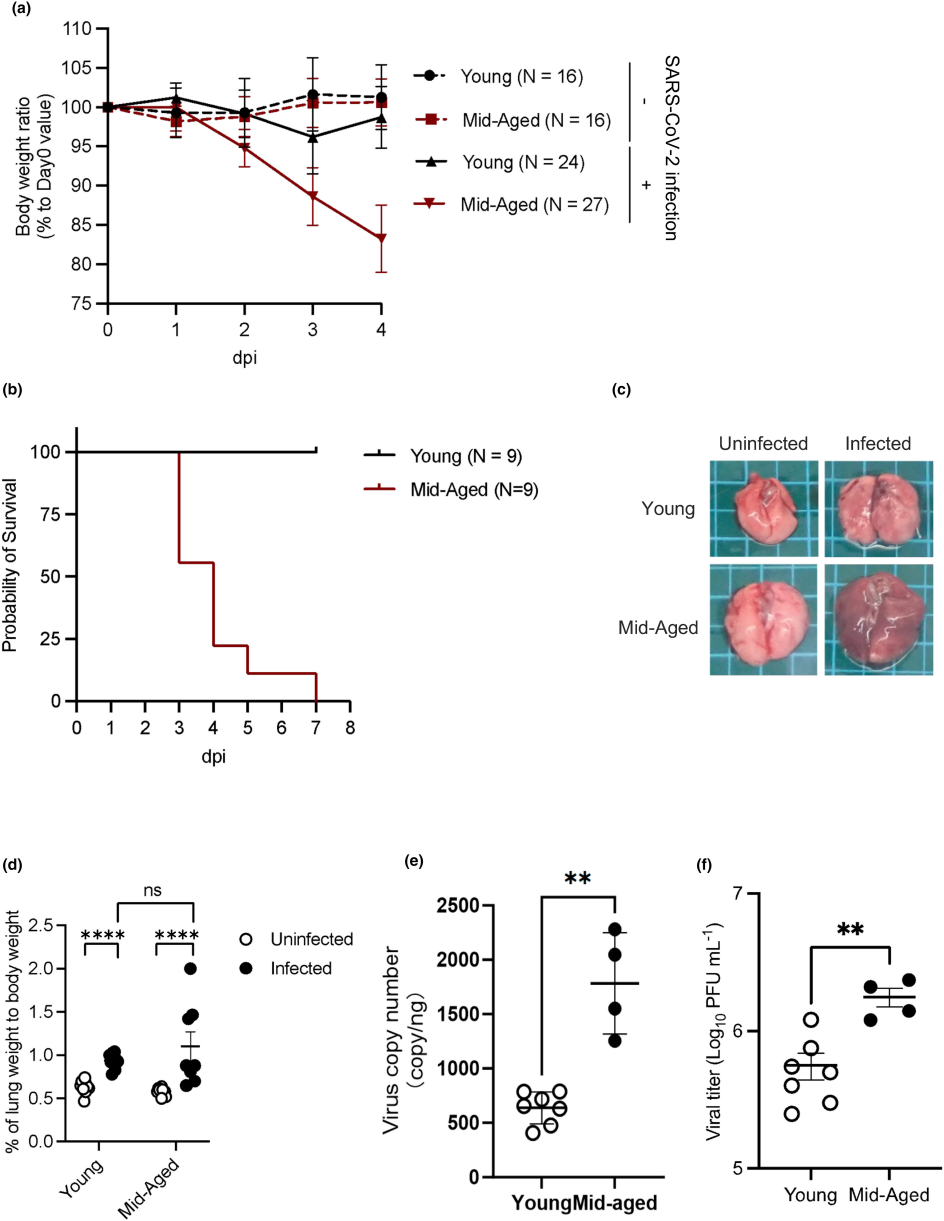
MA‐P10‐infected mice. (a) The body weights of young and mid‐aged BALB/c mice were monitored daily after intranasal administration of MA‐P10 SARS‐CoV‐2 (infected) or PBS (uninfected). Data are represented as a mean ± SD. dpi: days post infection (a, uninfected young: *n* = 16; uninfected mid‐aged: *n* = 16; infected young: *n* = 24; infected mid‐aged: *n* = 27). (b) Probability of survival of young and mid‐aged BALB/c mice after MA‐P10 SARS‐CoV‐2 inoculation (*n* = 9). (c) Representative gross appearance of the lungs of young and mid‐aged uninfected and infected mice at 4 dpi. (d) Relative lung weight to body weight of young and mid‐aged mice uninfected and infected at 4 dpi. The points indicate data from an individual mouse. Statistical analysis was performed using a Mann–Whitney test, where ****p < 0.0001; ns, not significant (uninfected young: *n* = 10; uninfected mid‐aged: *n* = 10; infected young: *n* = 11; infected mid‐aged: *n* = 8). (e) Virus copy number in the lungs of infected young and mid‐aged mice at 4 dpi. The points indicate data from an individual mouse. Statistical analysis was performed using a Mann–Whitney test, where **p < 0.01 (infected young: *n* = 7; infected mid‐aged: *n* = 4). (f) Viral titer in the lungs of infected young and mid‐aged mice, which were determined through plaque assay using the VeroE6/TMPRSS2 cells. Statistical analysis was performed using a Mann–Whitney test, where; **p < 0.01 (infected young: *n* = 7; infected mid‐aged: *n* = 4).

The BALB/c mice (female, young: 6 w/o, mid‐aged: 30–50 w/o) were inoculated intranasally with 1 × 10^4^ pfu of the mouse‐adapted SARS‐CoV‐2 (MA‐P10). The mid‐aged mice lost their body weight from 2 days postinfection (dpi) and died within 7 dpi without any weight recovery (Figure 1a,b). By contrast, the young mice showed slight body weight loss; however, their body weight recovered without lethal infections (Figure 1a,b). The lungs excised from the virus‐infected mice had red color, thus suggesting the existence of pulmonary inflammation (Figure 1c). The lungs of the mid‐aged mice specifically showed hepatization and increased body weights, indicating severe lung congestion (Figure 1d). The levels of viral RNA and infectious virus titer were significantly higher in the infected mid‐aged mice than in the infected young mice (Figure 1e,f).

### 2.2 Histopathological analysis of the mouse lungs

**FIGURE 2:**
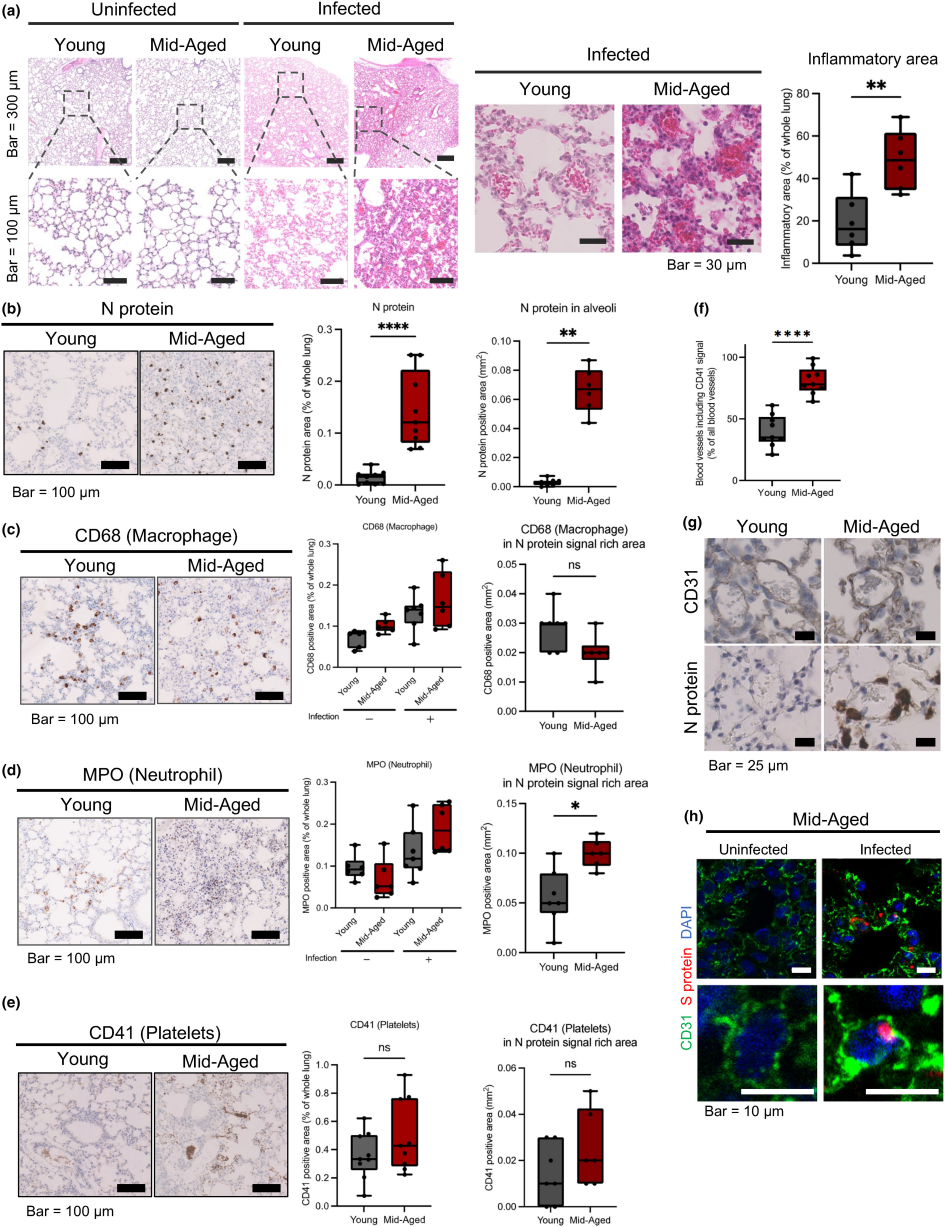
Pathological analysis of the lungs of MA‐P10‐infected‐mice. (a) (Left) Representative images of the mice lungs stained with H&E. Scale bars, top: 300 μm, bottom: 100 μm (Right). The rates of inflammatory areas in the lungs of infected young and mid‐aged mice at 4 dpi were quantified. Statistical analysis was performed using a Mann–Whitney test, where **p < 0.01 (infected young: *n* = 6; infected mid‐aged: *n* = 6). (b) Representative immunohistochemistry images of SARS‐CoV‐2 nucleocapsid protein (N protein) in the lungs of infected mice (left, Scale bar, 100 μm). The rate of N protein positive areas in the whole mice lungs and alveoli were measured (right). Statistical analysis was performed using a Mann–Whitney test, where ****p < 0.0001 (infected young: *n* = 9; infected mid‐aged: *n* = 9). (c) CD68 and (d) MPO in the mice lungs (left, Scale bar, 100 μm) and positive areas in the whole mice lungs or N protein positive area (right). Statistical analysis was carried out using a Mann–Whitney test, where *p < 0.05; ns, not significant. (e) Representative immunohistochemistry images of CD41 in the lungs of infected mice (left, Scale bar, 100 μm). The CD41‐positive areas in the whole mice lungs (middle) or in the N protein positive areas were measured (right). Statistical analysis was performed using a Mann–Whitney test. ns, not significant. (f) Rate of blood vessels including CD41 positive signals. Statistical analysis was accomplished using a Mann–Whitney test, where ****p < 0.0001 (Infected young: *n* = 9; infected mid‐aged: *n* = 9). (g) The representative serial section images of CD31 and N protein staining (middle, scale bar, 25 μm). (h) The representative immunofluorescence image of alveolar capillary vessels of CD31 and S protein staining (scale bar, 10 μm).

Histopathological analysis of the lungs of young and mid‐aged mice had been carried out at 4 dpi to examine the differences in pulmonary pathology of the infected mice and of the human cases of severe COVID‐19, as previously reported (Paul et al., 2022). Pulmonary hemorrhages, infiltration of the inflammatory cells, and vasculitis with perivascular accumulation of the inflammatory cells were observed only throughout the lungs of infected mid‐aged mice (Figure 2a). Notably, many thrombi were also observed in the mid‐aged group of mice (Figure 2a).

Immunohistochemistry showed that the SARS‐CoV‐2 nucleocapsid (N) signals in the whole lungs of the mice were significantly higher in the mid‐aged mice group than in the young mice group. Additionally, there was some difference in the alveolar area between the young and the mid‐aged mice groups (Figure 2b), which is consistent with the difference in viral loads recorded between the young and the mid‐aged mice groups (Figure 1e,f).

The CD68‐positive area observed in the whole lungs of the mice tended to be larger; however, it was smaller in the N protein signal‐rich area in the infected mid‐aged mice than in the young mice (Figure 2c). Infiltration of the MPO‐positive neutrophils was more evident in the mid‐aged mice (Figure 2d). The ratio of CD11b^+^Ly6G^+^ cells (Figure S1A) and CD11b^+^Ly6C^+^ cells (Figure S1B) in the whole lungs of the mice tended to increase in the infected mid‐aged mice group than in the young mice group, suggesting that more neutrophils and macrophages tended to infiltrate into the lung tissues of the mice upon viral infection. To detect the presence of thrombi in the virus‐infected lungs, a CD41 (platelet marker) immunostaining was performed. The number of vessels containing CD41‐positive platelet aggregates was significantly higher in the mid‐aged mice group than in the young mice group (Figure 2e,f), suggesting that thrombus formation had occurred in the infected mid‐aged mice, as it occurs in the human COVID‐19 cases. Additionally, immunohistochemistry of the mice lungs showed more N protein in the alveolar–capillary area of the mid‐aged mice than in the young mice. N protein signals were larger in mid‐aged mice than in young mice, suggesting increased infection of Type II alveolar epithelial cells or macrophages (Figure 2g). Furthermore, fluorescent double immunostaining of CD31 and SARS‐CoV‐2 spike (S) showed the presence of S protein signal surrounded by CD31 signal, which is expressed on the endothelial cell membrane, supporting the uptake of virus by pulmonary endothelial cells as shown in Figures 1, 2h, and Video S1.

### 2.3 Isolation of the pulmonary ECs from the SARS‐CoV‐2‐infected mice and transcriptome analysis

To address the pathology of the pulmonary ECs in severe COVID‐19 infection, the gene expression profile of ECs in the young and the mid‐aged mice that were infected with MA‐P10 was investigated. For RNA‐Seq analysis, the pulmonary ECs were isolated from the lungs of the young and the mid‐aged mice with/without the viral infection.

**FIGURE 3:**
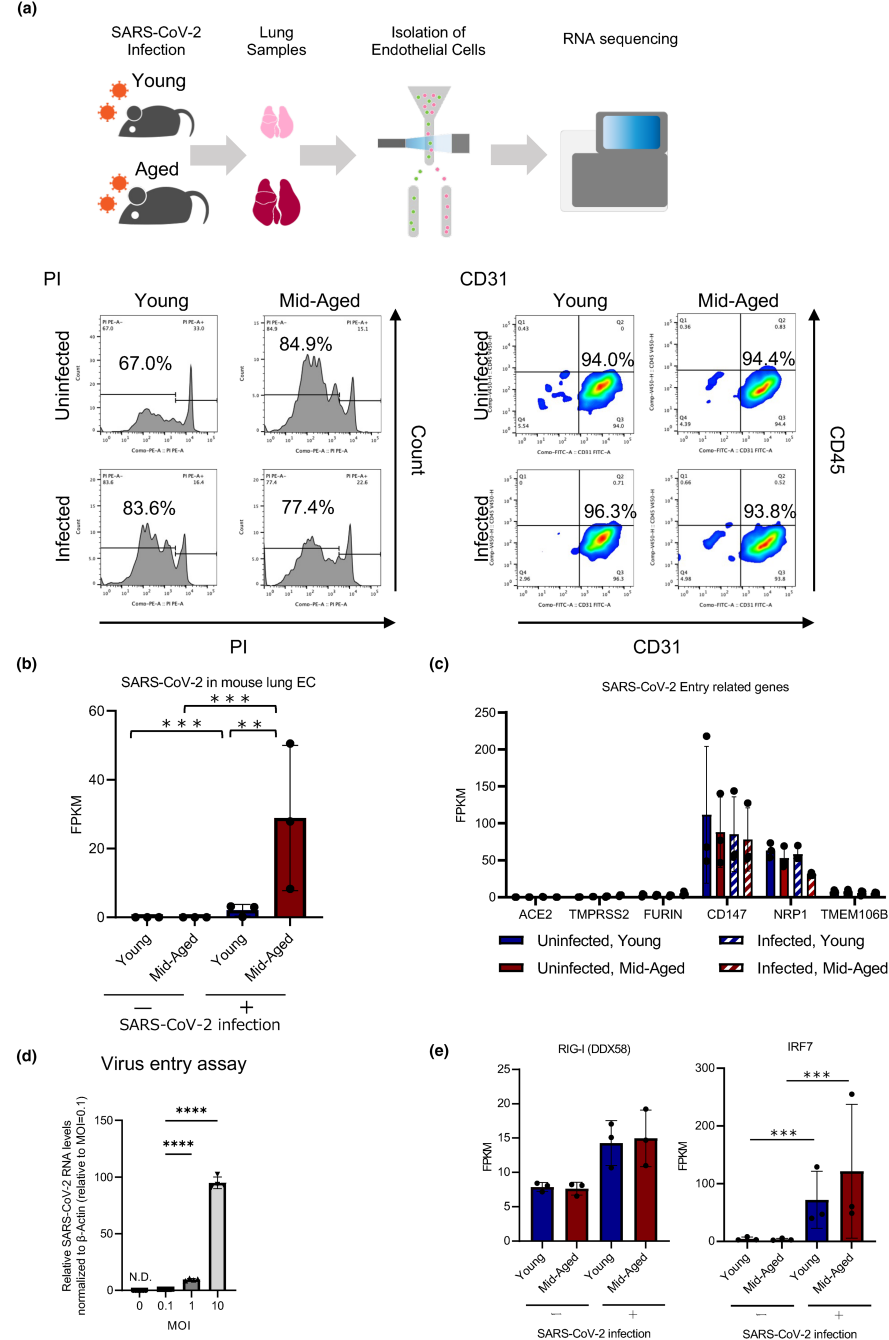
RNA‐Seq of the pulmonary endothelial cells of MA‐P10‐infected mice. (a), Schematic images of RNA sequencing (RNA‐Seq) of the pulmonary endothelial cells (ECs) of mice infected with MA‐P10 SARS‐CoV‐2, sacrificed at 4 dpi (upper), and sorting results of flow cytometry (lower). PI‐negative rate indicates viability, whereas CD31‐positive CD45‐negative rates indicate purity. (b) SARS‐CoV‐2 RNA expression levels in the pulmonary ECs achieved from the RNA‐Seq data, ***q‐value < 0.005, **q‐value < 0.01, and *q‐value < 0.05 (infected young: *n* = 3; infected mid‐aged: *n* = 3). (c) Expression levels of SARS‐CoV‐2 entry‐related genes (ACE2, TMPRSS2, furin, CD147, and NRP1, TMEM106B) that were achieved from RNA‐Seq data. (d) Relative SARS‐CoV‐2 RNA levels in HUVEC normalized to β‐actin. Statistical analysis was performed using a Dunnett's multiple comparison test, where ***p < 0.001. (e) Expression levels of DDX58 and IRF7 that were achieved from RNA‐Seq data. ***q‐value < 0.005, **q‐value < 0.01, *q‐value < 0.05.

The ECs were isolated as CD31^+^CD45^−^ cells from the lungs of 10 mice in each group, through a combination of a magnetic cell separation system (MACS) and a flow cytometric cell sorting, according to our previous method of EC isolation (Hida et al., 2004; Tsumita et al., 2022; Figure S3A). The recovered ECs from each group were 93%–96% CD31^+^CD45^−^, indicating high purity of the isolated ECs. Additionally, approximately 67%–84% of the isolated ECs were PI‐negative, demonstrating that the viability of the recovered ECs was enough to be used for subsequent RNA‐Seq (Figures 3a and S3B–D). The levels of viral RNA in ECs that were isolated from the lung tissues of the infected mice were quantified using quantitative reverse transcription polymerase chain reaction (qRT‐PCR). The results showed that the viral RNA was 14‐fold higher in ECs of the mid‐aged mice lungs than in ECs of the lungs of the young mice, suggesting that more viral uptake had occurred in ECs of the mid‐aged mice lungs (Figure 3b). The expression of the cluster of differentiation 147 (CD147) and neuropilin 1 (NRP1) among the molecules that were involved in SARS‐CoV‐2 entry was observed in all ECs, whereas the expression levels of ACE2 and TMPRSS2 (an EC surface protein) that represent the key molecules involved in viral uptake were low (fragments per kilobase million: FPKM < 10). Additionally, TMEM106B, as an alternative receptor for SARS‐CoV‐2 entry into ACE2‐negative cells, was expressed at very low levels (Figure 3c). These findings revealed that viral entry into the pulmonary ECs was independent on the ACE2 expression, which was different from the canonical route of the SARS‐CoV‐2 entry. In the pathological analysis, more viral antigens around ECs in the mid‐aged mice group were observed; thus, we investigated whether the cultured human umbilical vein ECs (HUVECs) were susceptible to viral infection and uptake or not. The levels of viral RNA in ECs increased in an MOI‐dependent manner, indicating that viral uptake had occurred in ECs in the form of an increase in the amount of viral exposure (Figure 3d). The results of RNA‐Seq of the mouse pulmonary vascular endothelium revealed that the expression levels of DDX58, a gene encoding the nucleic acid recognition sensors RIG‐I and IRF7 that was downstream of DDX58, were significantly upregulated in the infected mid‐aged mice group (Figure 3e). These findings suggest that SARS‐CoV‐2 was uptaken and stimulated an innate immune response in the vascular endothelium.

### 2.4 Upregulation of the inflammation‐related genes in the pulmonary ECs upon SARS‐CoV‐2 infection

**FIGURE 4:**
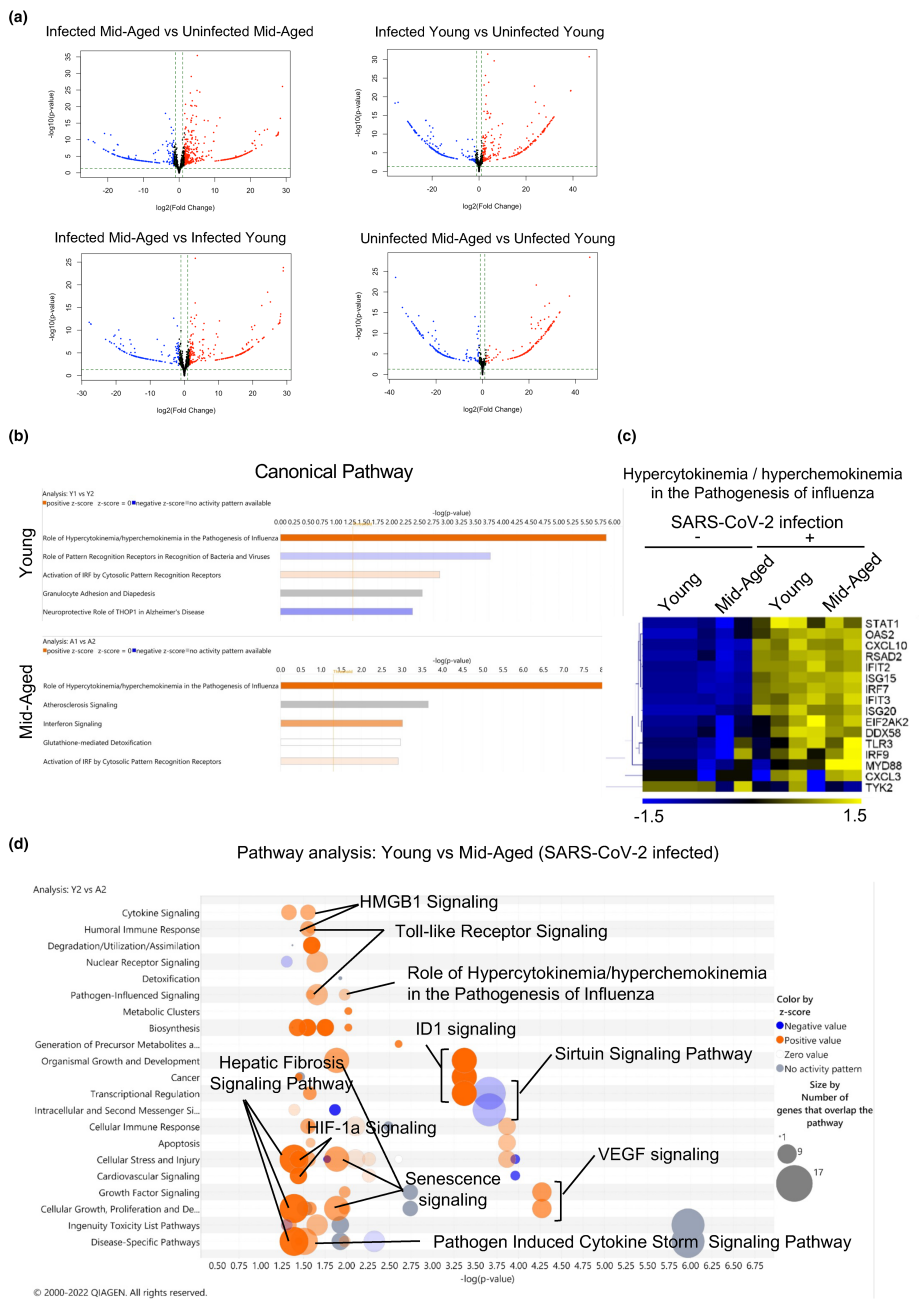
IPA analysis of RNA‐Seq data. (a) Volcano plots of the distribution of all differentially expressed genes between the infected mid‐aged versus uninfected mid‐aged, infected young versus uninfected young, infected mid‐aged versus infected young, and uninfected mid‐aged versus uninfected young mice. Red dots: upregulated genes, blue dots: downregulated genes. (b) Top five canonical pathways with the highest variability in each of the young and mid‐aged mice groups. (c) Heatmap demonstrating the expression levels of the genes that were related to the canonical pathway; with the highest variability “hypercytokinemia and hyperchemokinemia in the pathogenesis of influenza.” (d) Bubble chart exhibiting pathways with the highest variability, comparing the infected young and infected mid‐aged mice groups.

**FIGURE 5:**
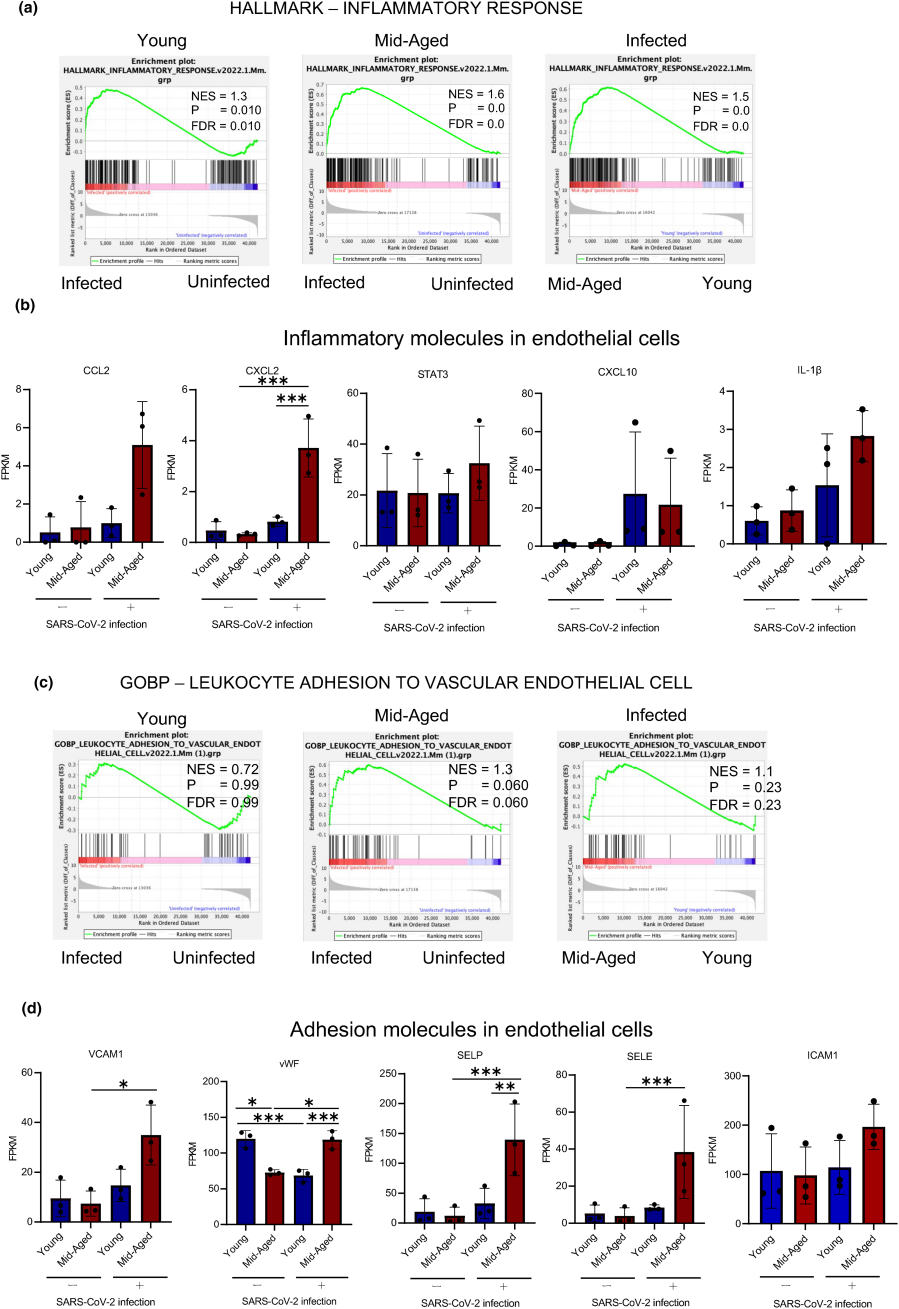
Inflammation of the pulmonary endothelial cells of MA‐P10‐infected mice. (a, c) The Gene Set Enrichment Analysis (GSEA) result of RNA‐Seq data of the pulmonary ECs of mice infected with MA‐P10 SARS‐CoV‐2, which were uploaded into GSEA for enrichment analysis of the inflammatory response (a), and leukocyte adhesion to the ECs (c). (b, d) Expression levels of representative molecules associated with each function that was achieved from RNA‐Seq data. ***q‐value < 0.005, **q‐value < 0.01, *q‐value < 0.05.

Differential expression analysis of individual genes was performed between pulmonary ECs from young mice and those from mid‐aged mice treated with PBS or SARS‐CoV‐2 using the DESeq2 package, following the criteria of an adjusted *p*‐value < 0.05 (Figure 4a). Comparing the effect of SARS‐CoV‐2 infection and the control, significant gene expression changes were found in the mid‐aged mice (upregulated: *n* = 910 genes, downregulated: *n* = 491 genes) compared with those in young mice (upregulated: *n* = 352 genes, downregulated: *n* = 241 genes). Comparing the mid‐aged and young mice, 485 significantly upregulated and 330 significantly downregulated genes were observed in the SARS‐CoV‐2‐infected groups, whereas 169 significantly upregulated and 178 significantly downregulated genes were detected in the uninfected group (Figure 4a). To recognize the canonical pathways and networks caused by SARS‐CoV‐2 infection of the pulmonary ECs in both the young and mid‐aged mice, the ingenuity pathway analysis (IPA) was applied. The graphical summary illustrated an outline of the connections between the key biological themes and the molecules in the IPA core analysis, which were regulated through SARS‐CoV‐2 infection. “Role of hypercytokinemia/chemokinemia in the pathogenesis of influenza,” is a common key of biological themes, which was upregulated by SARS‐CoV‐2 infection between the young and mid‐aged mice groups. This was accompanied by an enhancement of the Interferon‐regulated factor 3 (IRF3) and IRF7, which represent the two pivotal transcription factors that manage the production of IFN‐α and IFN‐β (Figure S4). The top five significant canonical pathways are listed for each of the young and mid‐aged mice groups (Figure 4b). Moreover, “role of hypercytokinemia/chemokinemia in the pathogenesis of influenza,” was the most significantly affected pathway by the viral infection in both young and mid‐aged mice groups, followed by the activation of the IRF through the cytosolic pattern recognition receptors (Figure 4b). Heatmaps of the differences in the expression levels of the genes listed as “role of hypercytokinemia/chemokinemia in the pathogenesis of influenza” between the four groups of ECs demonstrated that the expressions of *CXCL10* (C‐X‐C motif chemokine ligand 10), *EIF2AK2*, *DDX58*, and *IRF7* that were involved in the viral response were also highly expressed in the mid‐aged mice lung ECs (Figure 4c). The bubble chart expressed the predicted differences in the activity of the canonical pathway in ECs, which were recovered from the young or mid‐aged mice infected with SARS‐CoV‐2 (Figure 4d). These pathways were sorted according to the statistical significances and were colored according to the predicted activation status, which was indicated by a z‐score. The number of genes that overlapped each pathway was indicated by the sizes of the bubbles. Additionally, the bubble chart showed the inflammation‐related pathways. The role of hypercytokinemia/hyperchemokinemia in the pathogenesis of influenza, the pathogen‐induced cytokine storm signaling pathway, and the toll‐like receptor signaling were more activated in the mid‐aged mice than in the young mice. Besides the inflammation‐related pathways, the hypoxia‐related pathways, including HIF‐1a signaling, VEGF signaling, and hepatic fibrosis signaling, were also more enhanced in the mid‐aged mice than in the young mice (Figure 4d). The gene set enrichment analysis (GSEA) demonstrated that the genes in HALLMARK inflammatory response were more highly expressed in the mid‐aged mice group than in the young mice group (Figure 5a). The FPKM of CCL2, CXCL2, and CXCL10 in ECs was comparable among the four groups. The CXCL10 expression was significantly upregulated upon viral infection in both of the young and mid‐aged mice groups. However, the CXCL2 were significantly upregulated on viral infection in the mid‐aged mouse ECs. The proinflammatory cytokines such as interleukin‐1β (IL‐1β), which are related to the severity of COVID‐19, were not significantly different among the four groups. Conversely, the signal transducer and activator of transcription 3 (Stat3) that is activated by the JAK–STAT pathway downstream of IL‐6 was upregulated in the infected mid‐aged mice group (Figure 5b). The inflamed ECs are known to increase the expression of the adhesion molecules to the immune cells, including the leukocytes (Dayang et al., 2019). The GSEA showed that ECs in the mid‐aged mice group had more active “leukocyte adhesion to vascular endothelial cells” signaling than the young mice ECs (Figure 5c). In this regard, the expression levels of VCAM1, vWF (von Willebrand factor), P‐selectin (a type‐1 trans‐membrane protein), and E‐selectin (an adhesion receptor) increased significantly upon viral infection in the mid‐aged mice ECs. Alternatively, there was no significant difference in ICAM‐1 (intercellular adhesion molecule 1), although there was a tendency of upregulation of ICAM‐1 in the mid‐aged mice ECs (Figure 5d).

### 2.5 Upregulation of the coagulation‐related genes in ECs of mid‐aged mice on SARS‐CoV‐2 infection

**FIGURE 6:**
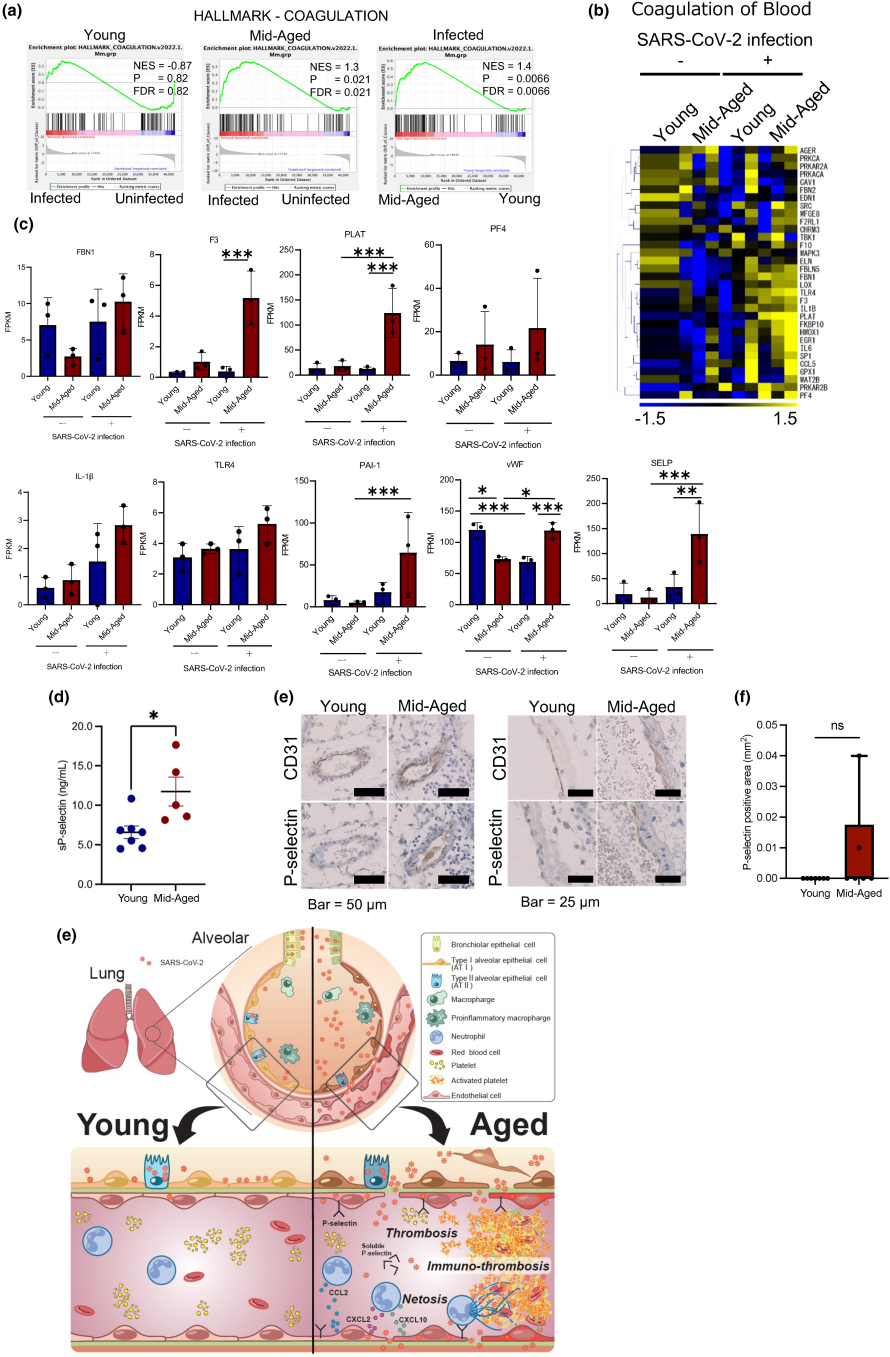
Thrombosis of the pulmonary endothelial cells of MA‐P10‐infected mice. (a) The GSEA results of RNA‐Seq data of the pulmonary ECs of mice infected with MA‐P10 SARS‐CoV‐2, which were uploaded into GSEA for enrichment analysis of the coagulation. Infected versus uninfected in the young, aged mice group (left), infected versus uninfected in the mid‐aged mice group (middle), infected young versus infected mid‐aged mice group (right), respectively. (b) Heatmap showing the expression levels of genes that were related to the “coagulation of blood” in QIAGEN coronavirus networks. (c) Expression levels of the representative molecules that were associated with blood coagulation and achieved from RNA‐Seq data. ***q‐value < 0.005, **q‐value < 0.01, *q‐value < 0.05. (d) Soluble P‐selectin concentration in the mouse plasma (infected young: *n* = 7; infected mid‐aged: *n* = 5 (e) Representative immunohistochemistry serial section images of the mice lungs for CD31 and P‐selectin). Statistical analysis was performed using a Mann–Whitney test, where *p < 0.05. (f) P‐selectin positive area in the N protein positive area. Statistical analysis was performed using a Mann–Whitney test. Ns, not significant. (g) Schematic image of the proposed pathogenesis in the pulmonary blood vessels of young and mid‐aged mice that were infected with MA‐P10 SARS‐CoV‐2.

The GSEA showed that “coagulation‐related genes” were more upregulated in the mid‐aged mice ECs than in the young mice ECs (Figure 6a). Using IPA, the expression of coronavirus network and the blood coagulation pathway genes were also recorded to be upregulated by a viral infection in the mid‐aged mice ECs (Figure S5). A similar result was obtained on comparative analysis between the young and the mid‐aged mice groups (Figure 6b). Thus, the IPA network involved in blood coagulation demonstrated that more molecules were activated in the virus‐infected mid‐aged mice group. The heatmap of the coagulation‐related genes represented the difference in the levels of gene expression among the four groups of mice ECs. The genes that were upregulated in the virus‐infected mid‐aged mice group included F3, PLAT, and others (Figure 6b). The FPKM levels of Fbn1, F3, PLAT, Pf4, IL‐1β, Tlr4, PAI‐1, vWF, and P‐selectin, which are the well‐known thrombogenic molecules, were higher in ECs of the virus‐infected mid‐aged mice group (Figure 6c). Notably, the expression levels of PLAT were significantly upregulated upon viral infection in the mid‐aged mice ECs. Moreover, the PLAT expression was significantly higher in the infected mid‐aged mice ECs than in the infected young mice ECs. Meanwhile, SERPINE1 (PAI‐1) and P‐selectin showed a significant increase on viral infection of the mid‐aged mice ECs only (Figure 6c).

The P‐selection and vWF were expressed in the initial phase of coagulopathy, unlike the PLAT, PAI‐1, and TF, which were upregulated in the late phase (fibrinolytic system). Moreover, the P‐selection and vWF have been suggested as biomarkers for severe COVID‐19 (Gorog et al., 2022). The results of enzyme‐linked immunosorbent assay (ELISA) analysis of the blood levels of the virus‐infected mice expressed higher levels in the mid‐aged mice group than in the young mice one (Figure 6d). Immunohistochemistry demonstrated P‐selectin‐positive ECs, which were identified in the N protein signal‐rich area of the mid‐aged mice lungs, thus suggesting that the virus‐uptaking ECs had activated the platelets in the mid‐aged mice group (Figure 6e,f).

## 3 DISCUSSION

Age is a risk factor for COVID‐19. Several reports have shown increased severity of SARS‐CoV‐2 infection in aged mice via transcriptome analysis of whole lung tissue (Zhang et al., 2021). For example, interferon and adaptive responses were impaired in aged mice compared to young mice (Chen et al., 2022), and senescent cells became hyperinflamed due to infection (Camell et al., 2021). However, vascular inflammation and thrombosis mechanisms observed in severe COVID‐19 have not been elucidated in these animal models. Our group also developed a mouse‐adapted SARS‐CoV‐2 infection model (Uemura et al., 2023), which has shown that only mid‐aged mice develop fatal pneumonia with thrombosis. Because we have identified vascular endothelial pathology by isolating endothelial cells (Tsumita et al., 2022), we were motivated to investigate pulmonary endothelial cell pathology by isolating ECs from infected mice. Given the enrichment of the EC population, we were able to unveil EC gene expressions of age‐related severe COVID‐19 using this mouse model. Because ECs are not a major component of pulmonary tissue, isolating them offers an advantage for transcriptome analysis. Our transcriptome analysis results for pulmonary EC revealed significantly higher levels of viral genes in ECs from mid‐aged mice, accompanied by upregulation of viral response genes. In addition, the thrombogenesis‐ and the inflammation‐related molecules were upregulated in the mid‐aged ECs upon viral infection. To the best of our knowledge, this is the first time that the transcriptomes of the isolated pulmonary ECs of the severe/nonsevere infected mice were compared.

Although viral infection of ECs in human autopsy tissues has been suggested as a mechanism for the development of thrombosis in COVID‐19 (Fosse et al., 2021), their detailed pathophysiology remains unclear due to the lack of animal model to study it.

Our study clearly demonstrated that SARS‐CoV‐2 was uptaken into the pulmonary ECs of the mid‐aged mice pneumonia lungs. The transcriptome results revealed that upon viral infection, the pulmonary ECs had a distinct thrombopathy phenotype in the mid‐aged mice, but not in the young mice (Figure 6g).

The pulmonary ECs uptake high levels of the virus in the mid‐aged mice. Furthermore, the viral response was observed through viral uptake in ECs, accompanied by an increased expression of the DDX58, IRF7, and other downstream genes, suggesting that ECs respond to the virus followed by several pathological changes. However, expressions of the ACE2 receptor and TMPRSS2 were extremely low in both of the young and mid‐aged mice ECs, in agreement with the previous reports (Hashimoto et al., 2022) that were carried out on ACE2 expression in ECs. Conversely, we have confirmed that our MA‐10 viral cells were transmitted via a mouse ACE2 (Figure S6). These results suggest that the ECs had a different viral uptake mechanism, which was independent of ACE2. Besides ACE2, NRP1 (the neuropilin 1), and CD147 (an immunoglobulin superfamily member) have been reported to be involved in the mechanism of SARS‐CoV‐2 infection (Junqueira et al., 2022). Although the NRP1 and CD147 were expressed in the isolated ECs; however, there was no difference in their expression between the young and the mid‐aged mice, suggesting that they may be unlikely to be involved in the severity of this viral disease. Meanwhile, the viruses can also be internalized into the mice cells through the clathrin‐mediated endocytosis, as reported previously for SARS‐CoV‐2 (Bayati et al., 2021). This indicates that the host cells can be infected if the amount of a virus present in the surrounding area is high. However, to elucidate the mechanism of endothelial viral uptake, a further future study is required.

Currently, several pathways related to the inflammatory response and leukocyte adhesion to ECs were activated in the mid‐aged pulmonary mice ECs through viral uptake. For example, CCL2 and CXCL2 cytokines that are neutrophil attractants adhesion molecule for the leukocyte (Akbar et al., 2022), and VCAM‐1 (vascular cell adhesion molecule 1; Folco et al., 2018) expressions were upregulated in the mid‐aged mice ECs through viral uptake. These results suggest that neutrophil activation by ECs may be responsible for the severe COVID‐19 pneumonia.

It has been reported that vascular ECs undergoing inflammatory changes in the tumor microenvironment secrete high levels of CCL2, which attracts neutrophils and activates them, suggesting that neutrophil activation takes place through the inflamed ECs as a new mechanism (Tsumita et al., 2022). The activated neutrophils originally release their own citrullinated nucleic acids, known as neutrophil extracellular traps (NETs), into the extracellular space to form a sticky network structure that captures the pathogens and eliminates them (Zuo et al., 2021). Additionally, NETs are known to be associated with thrombosis in patients with COVID‐19 (Kinnare et al., 2022; Radermecker et al., 2020). Thus, inflammation of endothelial cells is also one of mechanisms for thrombosis. Our results of the histological analysis showed that upon viral infection, the MPO‐positive activated neutrophils had infiltrated in the lungs of the mid‐aged mice more than those that had infiltrated the lungs of young mice. Despite the neutrophil mobilization in the lungs of the mid‐aged mice; however, they become severely ill and then died. The functions of the neutrophils may be altered in the virus‐infected mid‐aged mice. To investigate the changes that occur during the interaction between ECs and neutrophils, or the alteration of the inflammation response of ECs with aging, further studies are urgently needed.

In the present study, the vascular ECs were recorded in the mid‐aged mice, which also upon viral infection showed increased expression of the platelet adhesion molecules, platelet activation molecules, and factors that were involved in blood coagulation. The platelet activation state differed between the infected ECs of the mid‐aged mice and the young mice, suggesting that these platelets were activated by ECs. Interestingly, the expression of PAI‐1 (plasminogen activator inhibitor 1), PLAT, P‐selectin (a type‐1 trans‐membrane protein), and vWF was upregulated in the infected mid‐aged mice group. This finding is consistent with results of a previous study (Helms et al., 2020), which reported that 40% of COVID‐19 patients had thrombosis, besides several previous reports of thrombosis association with severe COVID‐19 disease (Gorog et al., 2022; Helms et al., 2020; Knight et al., 2022; O'Sullivan et al., 2020).

P‐selectin was significantly upregulated in the ECs of the mid‐aged mice upon viral uptake in particular. Histologically, P‐selectin expression was observed not only in the platelets but also in ECs. Additionally, the plasma level of P‐selectin was significantly higher in the mid‐aged mice. It has been reported that P‐selectin is expressed in the pathological ECs and is considered as a mechanism of thrombus formation (Guy et al., 2019). Moreover, it has been previously revealed that the circulating dimerized soluble P‐selectin promotes inflammation and coagulation (Panicker et al., 2017). Additionally, P‐selectin has been reported as one of the biomarkers of COVID‐19 severity (Abd El‐Ghani et al., 2022; Karsli et al., 2021), supporting that our mouse model reflects COVID‐19 pathogenesis. However, our present study has several limitations. It insufficiently revealed the molecular mechanism of thrombosis with aging. We infected cultured vascular endothelial cells with the virus in vitro to investigate the mechanism of endothelial pathology through virus uptake. However, most genes, including RIG‐I, in which their upregulation was observed in in vivo ECs, were not induced in cultured ECs by virus infection (Figure S7). These findings suggest that viral infection, host aging, and/or microenvironmental factors, including hypoxia and intercellular communication, are closely related to the acquisition of the thrombogenic phenotype in ECs. Furthermore, our transcriptome data were obtained from mice with 4 dpi, which in the case of the aged mice, represents the vascular endothelial pathology after a severe condition just before death. These resulted mainly from severe diseases, and it was difficult to determine the cause of the severe disease from our results. Further analysis through continuous sampling at different time point after infection is necessary to identify the endothelial cell molecules that trigger severe diseases in infected, aged hosts. We are currently analyzing transcriptome data from vascular endothelial cells over time.

A new mechanism of COVID‐19‐induced thrombosis has been considered, which involves the damage of mice ECs (vascular wall damage) through cytokines from the immune cells or through blood coagulation, which have been triggered by the production of a tissue factor (Rotoli et al., 2021). However, the current results revealed that the virus‐uptaking ECs have activated the platelets or the neutrophils, which results in a thrombus formation.

The molecules whose expression is upregulated only through viral uptake in the mid‐aged ECs are expected to be biomarkers for severe COVID‐19. Translational future research is required to elucidate these molecules.

## 4 MATERIALS AND METHODS

### 4.1 Experimental design

This study was designed to address whether the endothelial cells are infected by SARS‐CoV‐2 and endothelial pathophysiology using a mouse‐adapted SARS‐CoV‐2‐infected mouse model. Pulmonary endothelial cells were isolated and purified to compare their transcriptome between nonsevere/severe (young/mid‐aged) mice. Pathophysiology of the pulmonary endothelial cells upon virus infection was comprehensively analyzed using RNA sequencing.

### 4.2 Cell cultures

The African green monkey kidney‐derived Vero E6 cells (ATCC) were maintained in Dulbecco's modified Eagle's medium (DMEM) that was supplemented with 10% heat‐inactivated fetal bovine serum (FBS) (v/v) and then incubated at 37°C in a humidified atmosphere containing 5% CO_2_. The Vero E6 cells stably express the human type II transmembrane serine protease (VeroE6/TMPRSS2) cells, which were described previously by Sasaki et al. (2021). The HUVECs (Lonza) were maintained in EC growth medium‐2 (EGM‐2; Lonza). Finally, the mouse oral squamous cells (MOC1; Kerafast) were maintained in Iscove's modified Dulbecco's medium.

### 4.3 siRNA transfection

Three stealth small interfering RNA (siRNA) for the mouse ACE2 (mACE2) receptor was obtained through the BLOCK‐iT RNAi design program (Invitrogen). Transfection was accomplished using the lipofectamine RNAiMAX reagent (Invitrogen) for the MOC1 cells as previously reported (Torii et al., 2021). For siRNA targeting the mACE2, the used sequences were 5′‐CCAGGCAACCUUUCCUGCUAAGAAA‐3′ and 5′‐UUUCUUAGCAGGAAAGGUUGCCUGG‐3′. The stealth control siRNA (Invitrogen, Medium GC, sequence not available) was used as a control.

### 4.4 Animal experiments

All the applied animal experiments were approved by the Ethical Committee for Experimental Animal Care of Hokkaido University, Japan (approval number: 20‐0113). Approximately 6‐week‐old or 36‐week‐old female BALB/c mice that were obtained from CLEA Japan (Tokyo, Japan) or from SLC Japan (Shizuoka, Japan) were used in the present experiments. Challenge SARS‐CoV‐2 virus [1.0  ×  10^4^ pfu (plaque‐forming unit) 50 μL^−1^] was injected through intranasal instillation, which was diluted in a phosphate‐buffered saline (PBS), as previously described by Leist et al. (2020). At 4 dpi, the lungs were excised using a sterile scalpel after collecting the blood samples through a cardiac puncture following euthanasia. The treated mice that reached the endpoint before 4 dpi were included in Figure 1b only.

### 4.5 Viral preparation

The SARS‐CoV‐2 WK‐521 (EPI_ISL_408667) viral strain was kindly provided by Dr. Saijo (National Institute of Infectious Diseases, Tokyo, Japan). The mouse‐adapted SARS‐CoV‐2 strain (MA‐P10) was established from this SARS‐CoV‐2 WK‐521 virus stock as previously described (Uemura et al., 2023). All experiments involving the use of SARS‐CoV‐2 were performed in the Biosafety Level‐3 (BSL‐3) facility of Hokkaido University, in accordance with the institutional guidelines.

### 4.6 RNA isolation and amplification using qRT‐PCR

The total RNA was extracted from the inoculated mouse cells or lung tissues using TRIzol Reagent (Thermo Fisher Scientific) and RNeasy Mini Kit (QIAGEN). The extracted RNAs were subjected to quantification using qRT‐PCR analysis; with the THUNDERBIRD Probe One‐step RT‐qPCR Kit (TOYOBO). A primer probe sets for N2 (Takara Bio) was used. The endogenous expression level of β‐actin was quantified as an endogenous control with primer and probe sets for the nonhuman primate β‐actin (Overbergh et al., 2005) and the mouse β‐actin (Mouse ACTB Endogenous Control, 4352933E, Thermo Fisher Scientific). The levels of SARS‐CoV‐2 N gene were normalized to that of β‐actin. All qRT‐PCR assays were performed using the CFX96 Real‐Time PCR System (BioRad).

### 4.7 Viral entry assay

The HUVECs were inoculated with SARS‐CoV‐2 WK‐521 at different multiplicity of infection (MOI = 0, 0.1, 1, and 10). After incubation at 37°C for 24 h, the cells were washed with 1 × PBS, trypsinized, quenched in DMEM supplemented with 2% FBS, centrifuged (300 × *g*, 3 min, 4°C), washed again with 1 × PBS, and centrifuged, and finally, the cell pellet was lysed in a TRIzol Reagent (Thermo Fisher Scientific). The intracellular virus was analyzed quantitatively using real‐time PCR (RT‐PCR).

### 4.8 The viral plaque assay

The mouse lungs were homogenized in 3 mL of Hank's balanced salt solution by TissueRuptor II (QIAGEN), and centrifuged at 1000 × *g* for 5 min at 4°C. The supernatants were filtrated using 0.45‐μm Millipore syringe filter (Sartorius) and then serially diluted with DMEM containing 2% FBS. The diluted mixture was inoculated into the VeroE6/TMPRSS2 cells and incubated at 37°C for 1 h with agitation. After incubation, the cells were overlaid with 2% FBS DMEM containing 0.4% Bacto agar (Becton Dickinson). After 48 h of incubation at 37°C, the cells were fixed with 3.7% buffered formaldehyde overnight and then stained with 1% crystal violet. The number of plaques was enumerated, and the viral titer was calculated in pfu mL^−1^.

### 4.9 Histopathological analysis

The formalin‐fixed paraffin‐embedded lung tissue sections (4.25 μm in thickness) were stained with hematoxylin and eosin (H&E). An immunohistochemistry assay was employed using several primary antibodies against SARS‐CoV‐2 spike protein (mouse, GTX632604, GeneTex), SARS‐CoV‐2 nucleocapsid protein (rabbit, GTX635679, GeneTex), CD31 (rabbit, ab28364, Abcam), CD41 (rabbit, ab134131, Abcam), CD45 (rat, #103202, Biolegend), MPO (rabbit, ab9535, Abcam), CD68 (rabbit, ab125212, Abcam), and P‐selectin/CD62P (rabbit, ab255822, Abcam). The used secondary antibodies include Goat Anti‐Rabbit Immunoglobulins/HRP (P0448, Dako), Goat Anti‐Mouse Immunoglobulins/HRP (P0447, Dako), and Rabbit Anti‐Rat Immunoglobulins/HRP (P0450, Dako). The liquid DAB^+^ Substrate Chromogen System (K3468, Dako) was used for color development. DAB signal‐positive areas of CD41, CD45, MPO, and CD68 were quantified using an ImageJ software (NIH). The area with a particularly high (N) protein positive signal was defined as “N protein signal‐rich area,” and the positive areas of CD41, CD45, MPO, and CD68 were quantified using an ImageJ software (NIH).

### 4.10 Immunofluorescence imaging of mouse lung ECs

The formalin‐fixed paraffin‐embedded lung tissue sections (8 μm in thickness) were dewaxed, rehydrated, and heat‐treated for 30 min in 10 mM Tris/1 mM EDTA buffer (pH 9.0) and cooled to room temperature. After cooling, slides were washed in PBS and incubated with 0.5% H₂O₂ for 10 min at RT to inhibit endogenous peroxidases. After PBS wash, sections were incubated with 5% Goat serum in 1 × PBS for 1 h at room temperature and incubated overnight at 4°C with primary antibodies against SARS‐CoV‐2 spike protein (mouse, GTX632604, GeneTex) and CD31 (rabbit, ab28364, Abcam) in PBS containing 5% goat serum. For negative controls, sections were incubated without the primary antibody or mouse and rabbit isotype antibody controls. Sections were rinsed and incubated with Goat anti‐Mouse IgG (H + L) Highly Cross‐Adsorbed Secondary Antibody, Alexa Fluor™ 594 (Invitrogen, A‐11032) and Goat anti‐Rabbit IgG (H + L) Highly Cross‐Adsorbed Secondary Antibody (Invitrogen, A32731) for 1 h at RT. Nuclei were counterstained with DAPI (Dojin chemical). TrueVIEW Reagent (TrueVIEWTM Autofluorescence Quenching Kit, Vector Laboratories) was used according to manufacturer instructions to reduce autofluorescence. Sections stained with secondary antibodies alone showed no specific staining.

### 4.11 Confocal fluorescence imaging and three‐dimensional (3D) reconstruction

Confocal imaging of the lung tissue sections stained with immunofluorescence described above was performed using a Nikon confocal laser microscopy system (A1‐ECLIPSE Ti2; Nikon). An objective was used: PLAN APO l D (×40/NA = 0.95). Three laser lines at 407, 488 and 561 nm for excitation and three filter cubes at 400 nm/480 nm, 480 nm/560 nm, and 560 nm/640 nm for detection were used. The images with 0.31 μm/pixel of 1024 × 1024 pixels, and a 12‐bit color depth were acquired. For detailed 3D fluorescence morphometry, confocal images with × 5 optical zoom were taken with 0.5 μm step sizes for voxel sampling. The image size of x‐y plane was 0.06 μm/pixel of 1024 × 1024 pixels. The 3D fluorescence images were constructed from z‐series images using the IMARIS software program (Bitplane) as described previously (Sci Rep. 2022 Oct 7;12(1):16799. doi: 10.1038/s41598‐022‐20,793‐5).

### 4.12 Isolation of the mouse lung ECs

The ECs were isolated from the mouse lung tissues following the protocol described previously (Cong et al., 2021; Hida et al., 2004; Kikuchi et al., 2020), with slight modifications.

Briefly, the excised lung tissues were minced using a sterile scalpel and then digested with collagenase II (Thermo Fisher Scientific) for 1 h at 37°C. After incubation, the blood cells were removed using a lysing buffer (BD Biosciences). The lung cell suspensions were then incubated at 4°C for 10 min with FcR blocking reagent (Miltenyi Biotec), followed by CD31 microbeads (Miltenyi Biotec). The CD31‐positive cells were isolated as lung ECs using a magnetic cell separation system (MACS; Miltenyi Biotec), according to the manufacturer's instructions. To improve the purity of the lung ECs, the cells were further sorted from a CD31^+^CD45^−^ population with Alexa Fluor 488‐conjugated anti‐mouse CD31 rat antibody (#102414, Biolegend) and Pacific Blue‐conjugated anti‐mouse CD45 rat antibody (#103125, Biolegend), through a flow cytometric cell sorter (FACS Melody; BD Biosciences). The data were acquired using a FACSMelody™ Cell Sorter and a BD FACSChorus™ Software (BD Biosciences) and then analyzed using a FlowJo software (Treestar).

### 4.13 The flow cytometric analysis of the mouse lungs

The flow cytometric analysis was performed on the lung cell suspensions, as indicated in “isolation of the mouse lung ECs.” The used antibodies included Brilliant Violet 510™ anti‐mouse/human CD11b rat antibody (Clone M1/70; #101245, Biolegend), Alexa Fluor® 488 anti‐mouse Ly‐6G rat antibody (#127626, Biolegend), and Alexa Fluor® 488 anti‐mouse Ly‐6C rat antibody (#128022, Biolegend).

### 4.14 Extraction and quality assessment of the RNA

The purified lung ECs were centrifuged (800 × *g*, 20 min, at 4°C), and the total RNA was extracted using a TRIzol™ LS (Thermo Fisher Scientific) and a RNeasy Mini Kit (QIAGEN). The quality of the extracted RNA was assessed using RNA 6000 Nano Assay kit and Agilent 2100 Bioanalyzer (Agilent Technologies). The RNA integrity number was generated and analyzed using a Bioanalyzer 2100 Expert Software (Agilent Technologies).

### 4.15 RNA‐Seq

RNA‐Seq was conducted three times independently (Exp. 1, 2, and 3). For Exp. 1, the sequencing library for RNA‐Seq was prepared using a Smartseq v4 Ultra Low Input Kit (Takara Bio) and Nextera XT DNA Library Prep Kit (Illumina) and then subjected to deep‐sequencing with NovaSeq 6000 (Illumina). For Exp. 2 and 3, the sequencing libraries were prepared with a SmartSeq Stranded Kit (Takara Bio) and then subjected to deep‐sequencing with NovaSeq 6000 (Illumina).

Read data were aligned to the mouse GRCm39 reference genome using STAR v2.7.10b (Dobin et al., 2013) with—quantMode Transcriptome SAM and—outSAM type BAM SortedByCoordinate option parameters. Assembling transcripts, merging assembled transcripts, and generating read coverage tables were performed using StringTie v2.1.5 (Pertea et al., 2015). Read coverage tables were converted to a DESeq2‐compatible form using prepDE.py3. Differential expression gene analysis was performed using DESeq2 v1.38.3 (Love et al., 2014) following standard protocols. The StringTie output file was used to count the data, which were tagged with sample group names and batch numbers. The DESeqDataSet object was constructed with ~batch + sample_group option in the design argument to remove batch effects. Differential expression gene analysis between each sample group was performed. The log‐fold change shrinkage function was applied for gene ranking and data visualization. The EnhancedVolcano package v1.16.0 (Blighe et al., 2023) was used to generate volcano plots. We constructed custom reference genomes for SARS‐CoV‐2 viral titer measurements by concatenating the SARS‐CoV‐2 WK‐521 (EPI_ISL_408667) sequence with the mouse genome (GRCm39). Before the FPKM calculation, a modified StringTie merged.gtf file was obtained with unique_gene_id.py (Boyle, 2020) to avoid gene ID conflict. The FPKM calculation was performed using Ballgown v2.30.0 (Frazee et al., 2015).

### 4.16 Pathway and network analysis

The IPA (QIAGEN) is a web‐based software application that was used to assess whether the molecules or the pathways of coagulation and inflammation (generated through use of QIAGEN IPA by QIAGEN Inc., Hilden, Germany, https://digitalinsights.qiagen.com/IPA) were activated or inhibited in the lung ECs of the infected mid‐aged mice, which were related to SARS‐CoV‐2 severity. The Ensemble ID number, the absolute TPM values from RNA‐Seq data (Exp. 1), and the log fold change value were imported into the IPA. The IPA core analysis was first carried out to identify the significantly affected candidate pathways. The significance of association between the gene expression data and the canonical pathways was determined by estimating the ratio of the number of overlapping genes from our dataset to the total number of IPA dataset genes in a particular pathway (Kramer et al., 2014). Afterward, the significance of association between the genes and the canonical pathway was statistically determined using the Fisher's exact test, where *p* < 0.05 was considered significant (QIAGEN IPA by QIAGEN Inc., https://digitalinsights.qiagen.com/IPA). The result of canonical pathway analysis was illustrated as a graphical abstract, the top five pathways list, and the bubble charts, respectively, to visualize the pathways that were related to the severity of the SARS‐CoV‐2 infection. A log2 fold change and absolute TPM counts were used for filtering the dataset.

Besides the IPA analysis and in order to identify and provide a visual representation of the deferentially expressed genes in the determined canonical pathways that were affected by SARS‐CoV‐2 infection, the hierarchically clustering was carried out using the MeV tool (Howe et al., 2011). The results were exported as a heatmap. For the hierarchically clustering analysis, the log Ratio calculated from TPM values were used (Makondi et al., 2018). The list of gene set that was used for the hierarchically clustering was derived from those in the IPA canonical pathway.

### 4.17 GSEA

The GSEA was employed using the GSEA software version 2.0.13, which was available from the Broad institute website (www.broadinstitute.org/gsea/). The hallmark gene sets were downloaded from the MSigDB database (Subramanian et al., 2005). All gene set files for this analysis were obtained from the GSEA website (www.broadinstitute.org/gsea/). An enrichment map was used for visualization of the GSEA results.

### 4.18 ELISA

The mouse plasma was obtained from the supernatant of the centrifuged heparinized whole blood, which was collected under isoflurane anesthesia. The concentration of P‐selectin (CD62P) in the plasma of each mouse was determined using a sandwich ELISA through the mouse P‐Selectin ELISA Kit (CD62P; ab200014, Abcam), according to manufacturer's instruction.

### 4.19 Statistics

The statistical analyses were performed using GraphPad Prism v9 (GraphPad Software Inc). Data are presented as the mean values ± standard deviation (SD) of the biological assays, which were carried out in triplicates. Statistical analysis of the data with a parametric distribution was carried out using one‐way analysis of variance. This was followed by a Dunnett's multiple comparison test for the multiple comparisons and or a two‐sided Student's *t* test for comparison between each two groups. However, the data with a nonparametric distribution was analyzed using a Mann–Whitney *U* test between each two groups. For all these data sets, a *p* value of less than 0.05 was considered significant. Meanwhile, ****p < 0.0001, ***p = 0.0001–0.001, **p = 0.001–0.01, and *p = 0.01–0.05 were considered significant.

The RNA‐Seq data analysis was performed using DESeq2, as described above. The Wald test was performed to identify differentially expressed genes between the two samples, and the Benjamini–Hochberg false discovery rate (FDR) was calculated for multiple test correction as adjusted *p*‐values. For the RNA‐Seq data analysis, ***adjusted p < 0.005, **adjusted p = 0.005–0.01, and *adjusted *p =* 0.01–0.05 were considered significant.

## AUTHOR CONTRIBUTIONS

Conceptualization: TT, RT, YH, KH, HS. Data curation: TT, RT, TI, HN, HA. Formal analysis: TT, RT, WI, TT, AM, TN, RZ, JWL. Methodology: TT, RT, NM, MS, YO, AS, ST, AM. Investigation: TT, RT, NM, YH, TI, HN, WI, TT, YS, AM, TN, RZ. Resources: NM, MS, YO, AS, ST, AM, HS. Visualization: TT, RT, WI, TT, YS, HW, TI. Supervision: KH, HS. Writing—original draft: RT, KH. Writing—review and editing: RT, NM, YH, MS, YO, JWL, TI, HS, KH.

## FUNDING INFORMATION

The Japan Agency for Medical Research and Development (AMED) grant 20fk0108537h0001 (KH) The Japan Agency for Medical Research and Development (AMED) grant JP223fa627005 (HS).

## CONFLICT OF INTEREST STATEMENT

The authors declare no competing interests.

## Supporting information


Figure S1‐S7.

Video S1.

Data S1.

## Data Availability

All data are available in the main text or the supplementary materials. RNA‐seq data were deposited in the Gene Expression Omnibus (GEO) database under accession number GSE230022.
