# Supplementary material for: Viral uptake and pathophysiology of the lung endothelial cells in age‐associated severe SARS‐CoV‐2 infection models
Source: Aging Cell. 2023 Dec 14;23(2):e14050. doi: 10.1111/acel.14050 (PMC10861199; doi:10.1111/acel.14050)
Supplement: Supplementary file 1 — Figure S1‐S7. [file ACEL-23-e14050-s003.pdf]

Supplementary fig.1

A

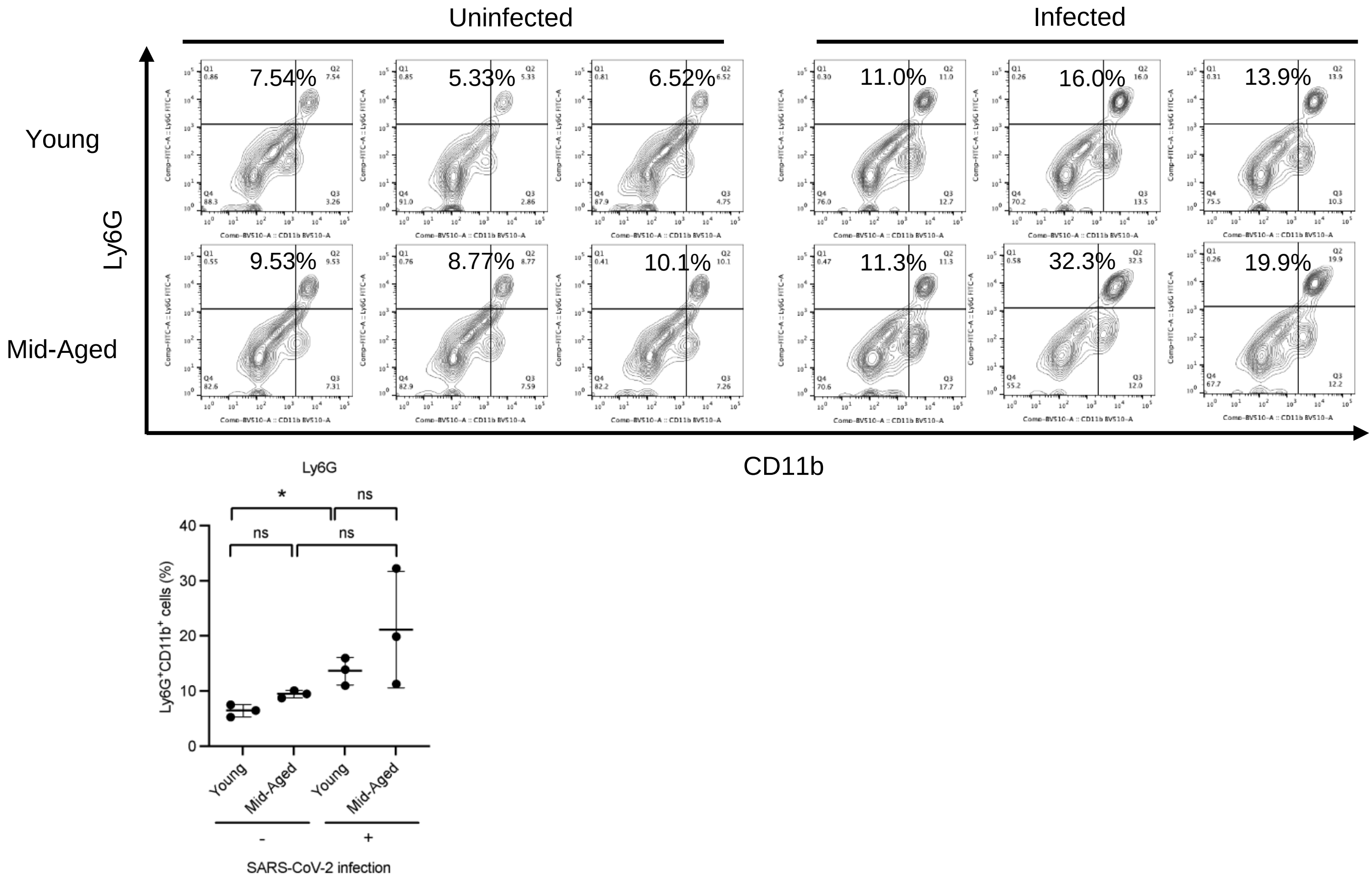

B

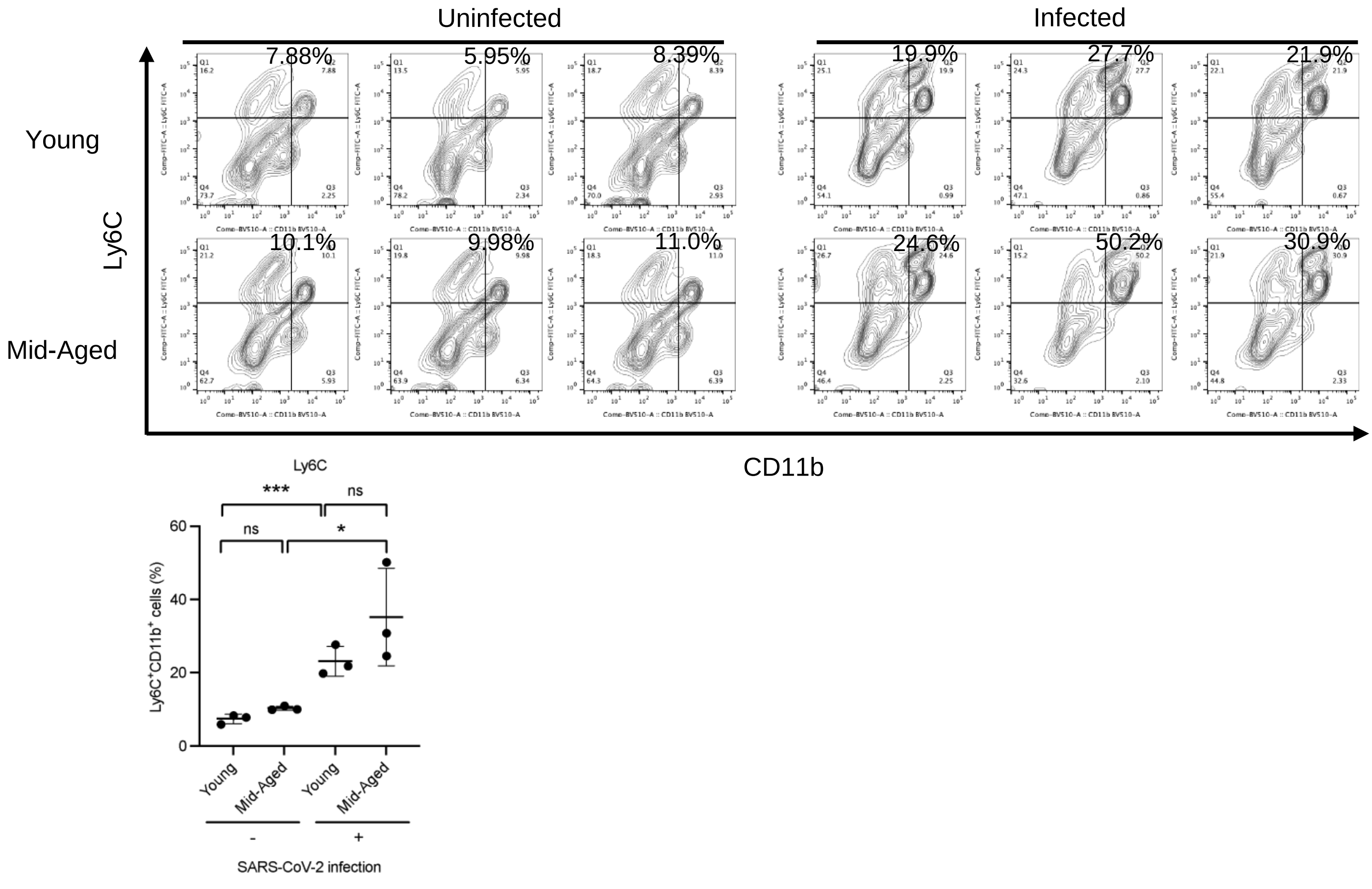

Supplementary fig.2

Mid-Aged
Infected

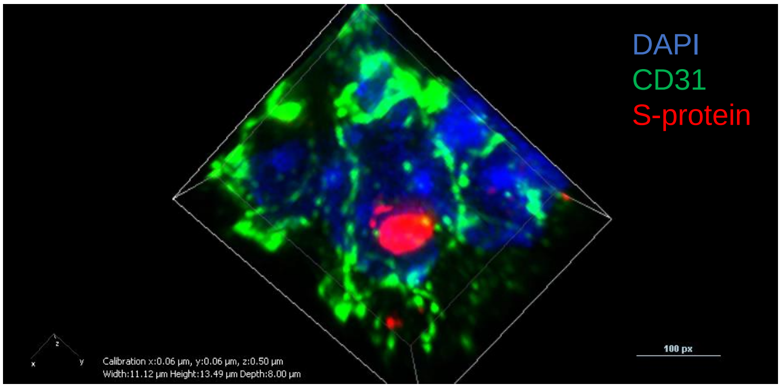

Supplementary fig.3

A

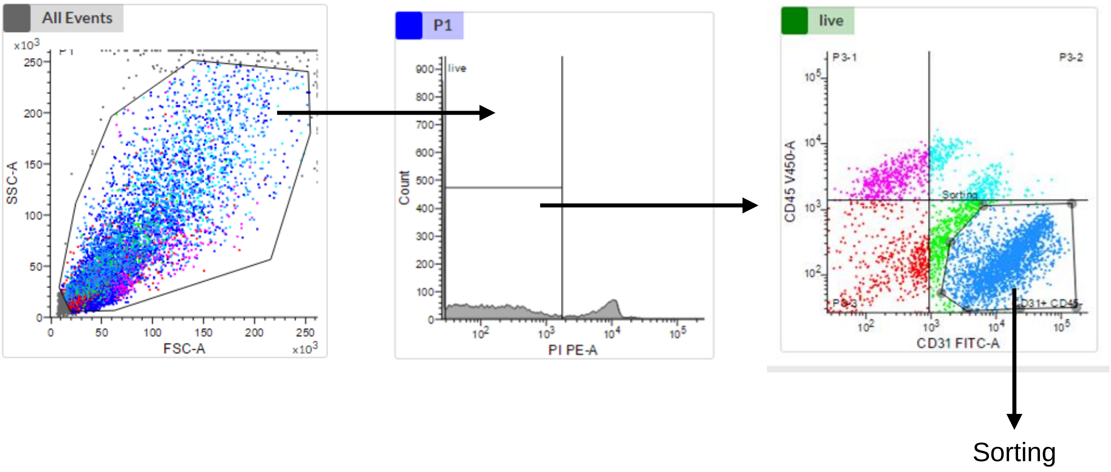

Supplementary fig.3

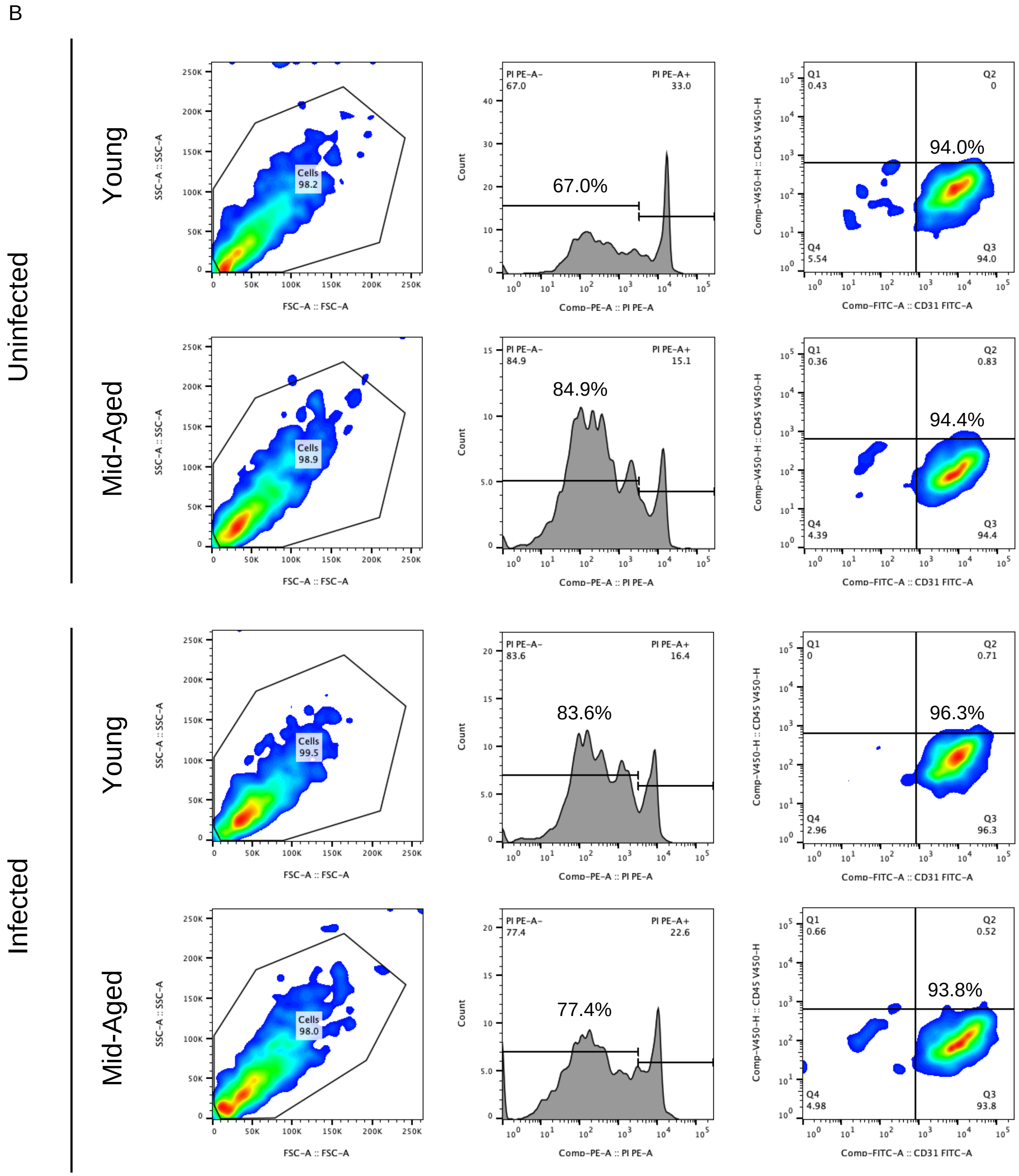

Supplementary fig.3

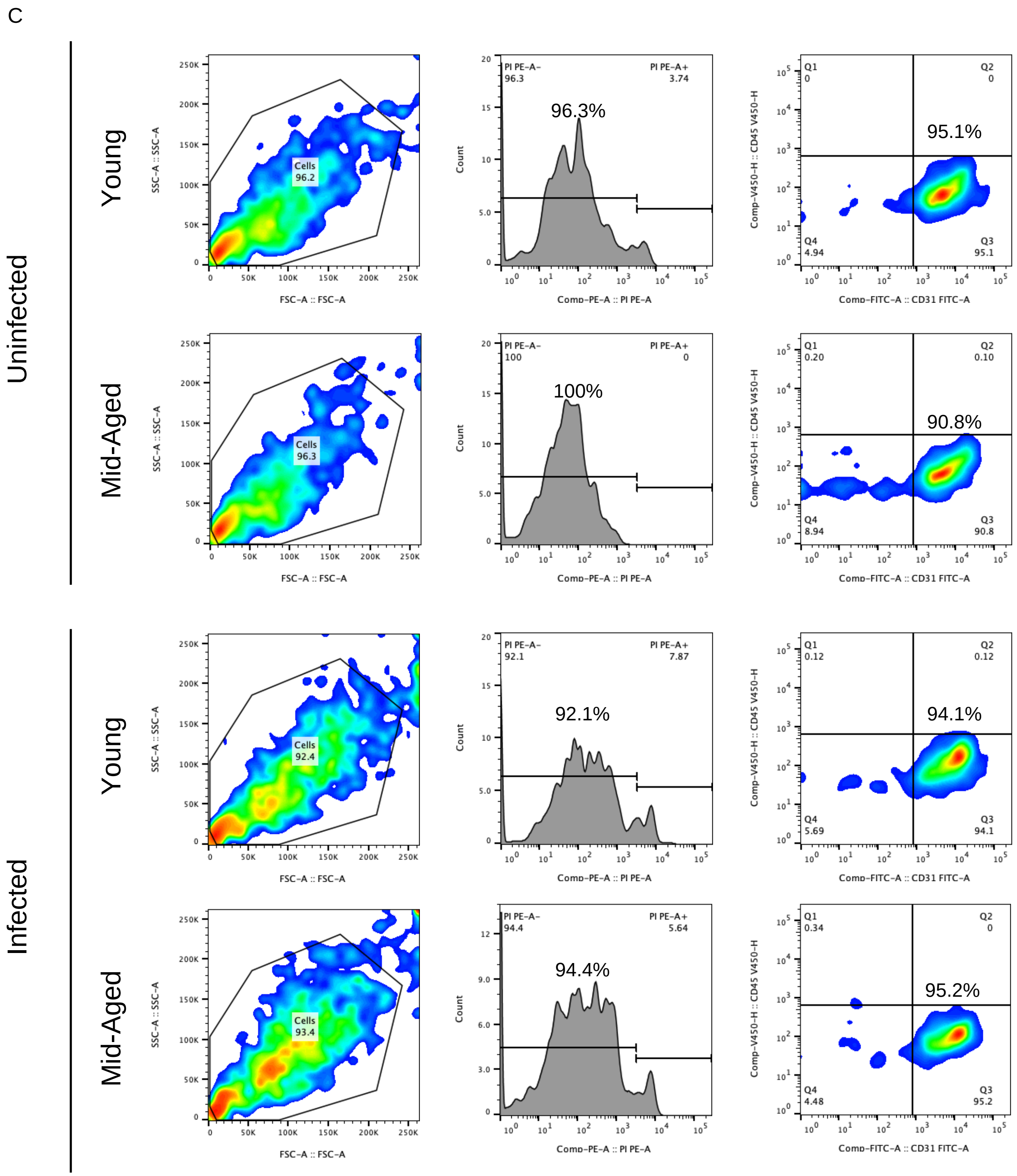

Supplementary fig.3

D

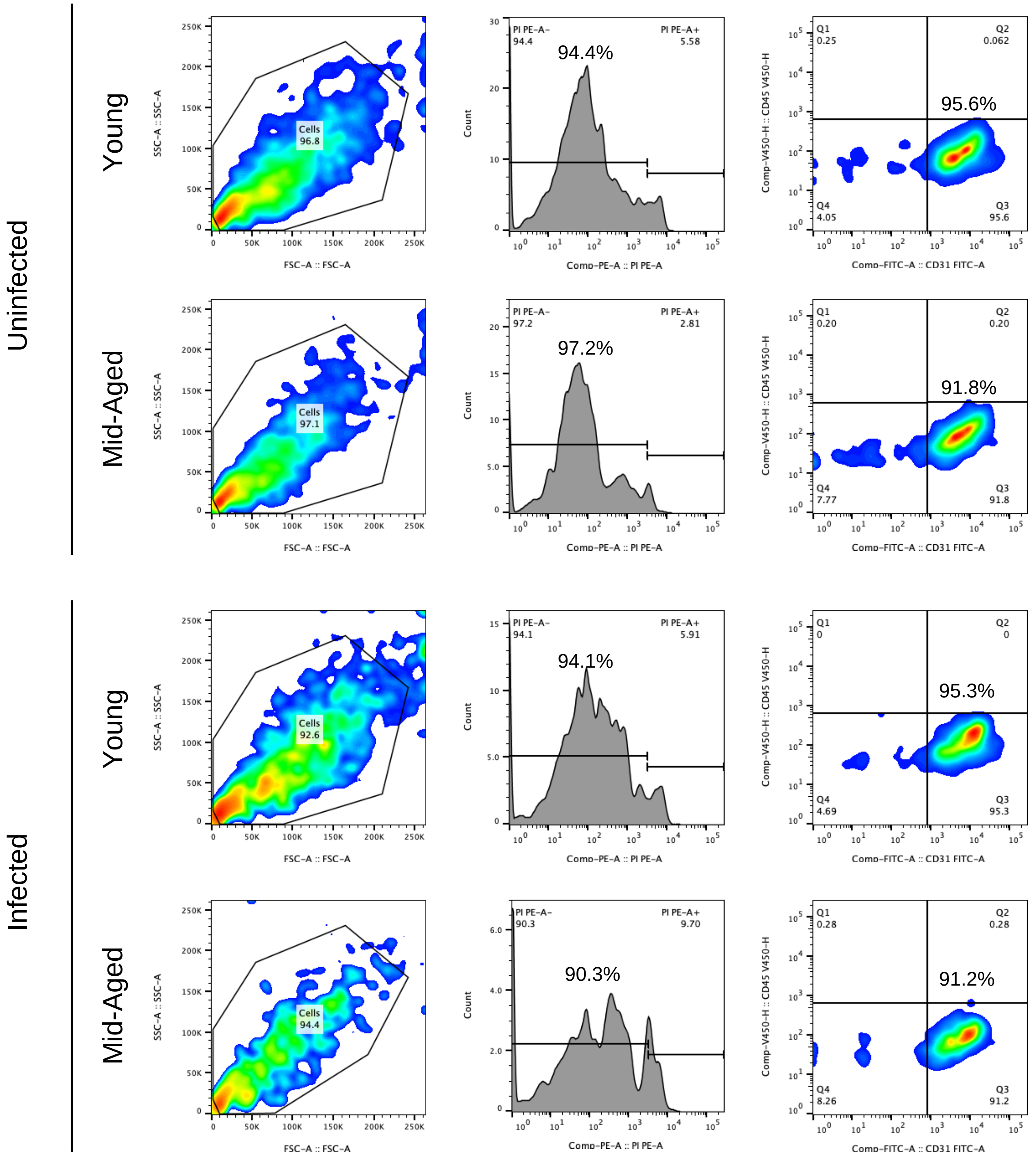

Supplementary fig.4

Fig. 4Aからサプリへ移動

Young

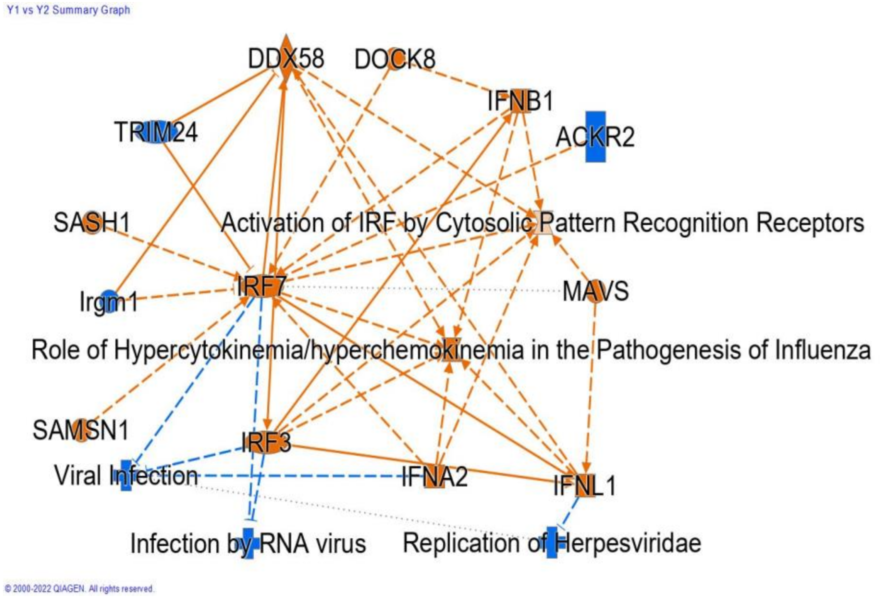

Mid-Aged

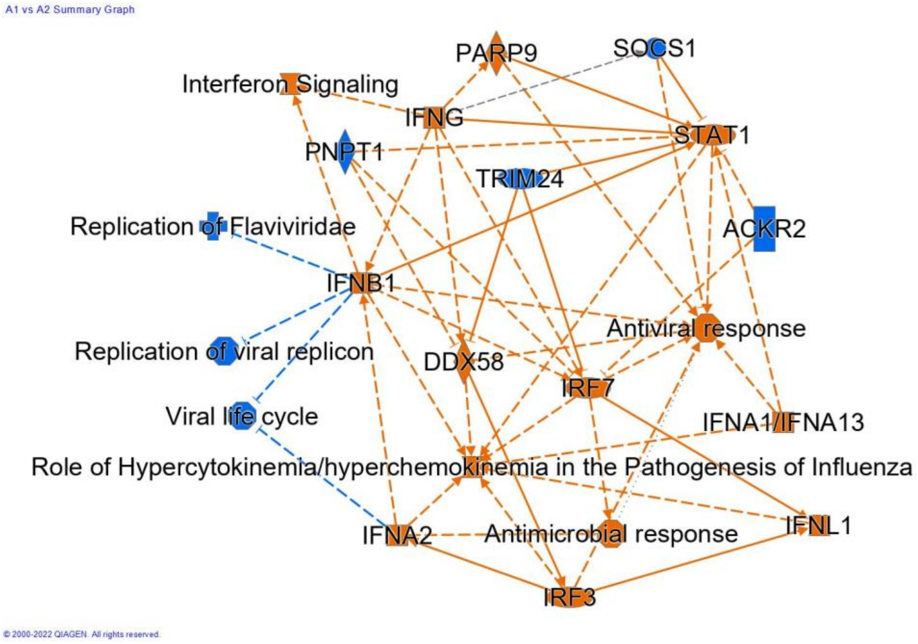

Supplementary fig.5

Fig. 6Bからサプリへ移動

Young

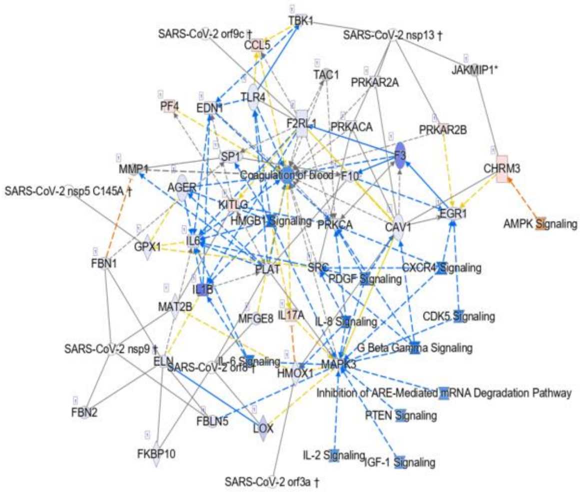

Mid-Aged

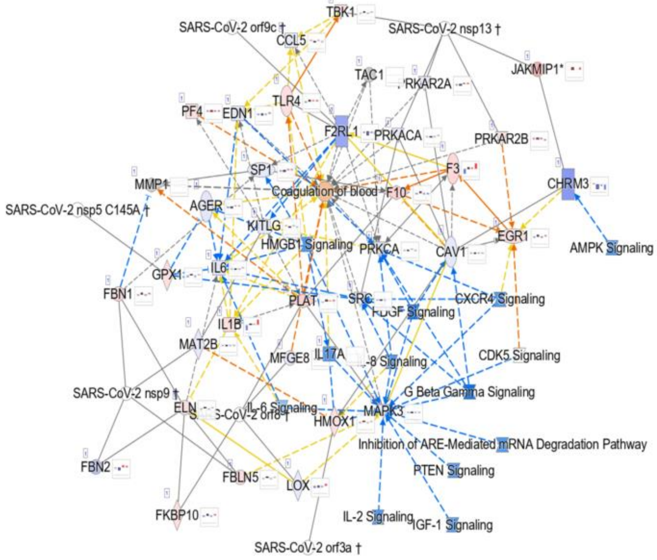

Young vs Mid-Aged

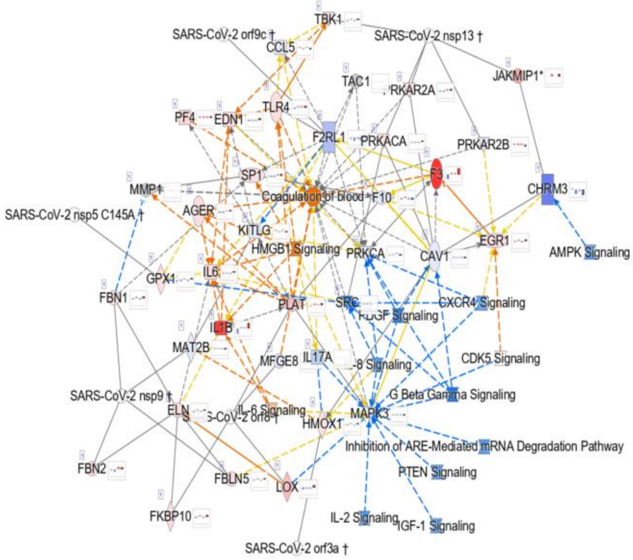

QIAGEN Coronavirus Networks - Coagulation of blood

Supplementary fig.6

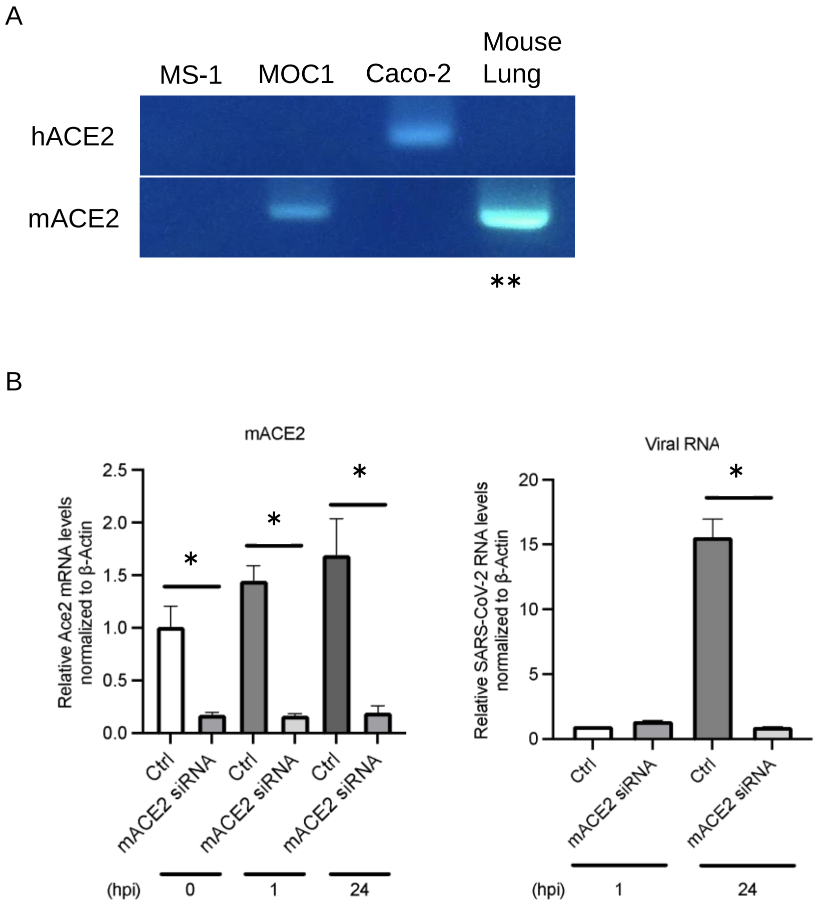

Supplementary fig.7

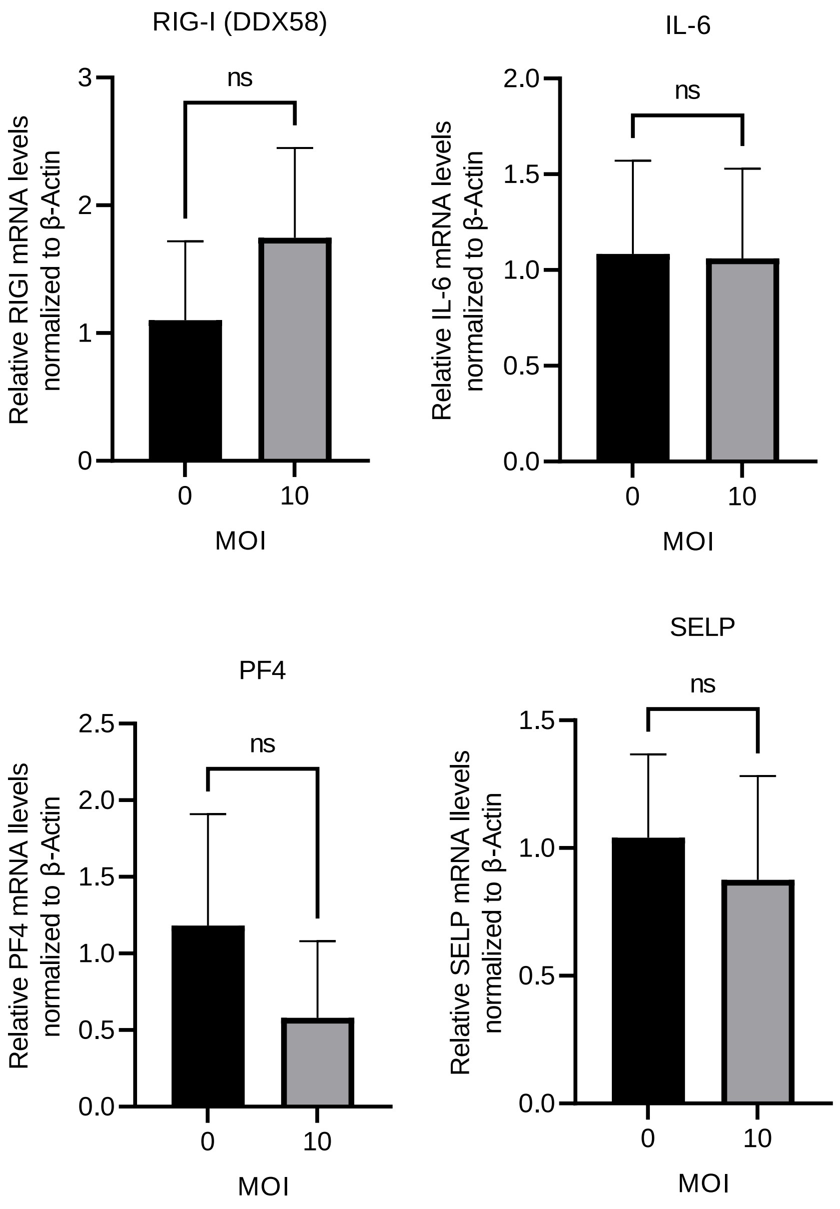

Supplementary fig. 1: CD11b$^+$ Ly6G$^+$/Ly6C$^+$ cell population in mice lung. (A), The CD11b$^+$ Ly6G$^+$ cell populations in the lungs of young aged and mid-aged mice, which were infected with MA-P10 SARS-CoV-2 and those that were uninfected (n = 3), for the flow cytometry analysis. **(B),** The CD11b$^+$ Ly6C$^+$ cell populations in the lungs of young aged and mid-aged mice, which were infected with MA-P10 SARS-CoV-2 and those that were uninfected (n = 3), for the flow cytometry analysis. Statistical analysis was accomplished using a Dunnett's multiple comparison test, where ***P = 0.0001 to 0.001, *P = 0.01 to 0.05; ns, not significant.

Supplementary fig. 2: The representative Immunofluorescence image of alveolar capillary vessels of CD31 and S protein staining.

Supplementary fig. 3: FACS gating strategy and soring results. (A), A Fluorescence-activated cell sorting (FACS) Gating strategy to isolate the ECs from lungs of mice. Side versus forward scatter plot showing gate P1 around the cells and excluding the cell debris. After the exclusion of dead (PI$^+$) cells, the live CD31$^+$ CD45$^-$ cells were isolated. **(B), (C), (D),** Sorting results of the isolated ECs in experiments 1, 2, and 3, respectively.

Supplementary fig. 4: Networks generated through the Ingenuity Pathway Analysis (IPA) for young and mid-aged mouse groups. The pathways and molecules activated in the infected group compared with the uninfected group are presented in orange, and those that were suppressed are presented in blue.

Supplementary fig. 5: Networks generated through IPA. Networks generated for young and mid-aged mouse groups, corresponding to figure 6A in each mouse group.

Supplementary fig. 6: mACE2 knockdown in MOC1 reduced susceptibility to MA-P10 SARS-CoV-2. (A), Results of polymerase chain reaction (PCR) showing a hACE2 positive band in Caco-2, mACE2 positive bands in MOC1, and in the mice lungs. **(B),** Results of quantitative PCR demonstrating the success of mACE2 knockdown in MOC1 (left), and it decreased the SARS-CoV-2 infection at 24 hpi (right). Statistical analysis was performed using unpaired *t*-test, where *$P < 0.05$.

Supplementary Fig. 7: *In vitro* gene expression analysis in infected human endothelial cells (HUVECs). HUVECs were infected by SARS-CoV-2. Expression levels of the representative molecules associated with virus response, inflammatory

response, and blood coagulation were analyzed at 24 hpi.
