# Supplementary material for: Viral uptake and pathophysiology of the lung endothelial cells in age‐associated severe SARS‐CoV‐2 infection models
Source: Aging Cell. 2023 Dec 14;23(2):e14050. doi: 10.1111/acel.14050 (PMC10861199; doi:10.1111/acel.14050)
Supplement: Supplementary file 3 — Data S1. [file ACEL-23-e14050-s001.docx]

The caption:

Supporting information Video S1 : The representative immunofluorescence image of alveolar capillary vessels of CD31 and S protein staining .
